# Exposure to sodium channel-inhibiting drugs and cancer survival: protocol for a cohort study using the QResearch primary care database

**DOI:** 10.1136/bmjopen-2014-006604

**Published:** 2014-11-14

**Authors:** Caroline Fairhurst, Ian Watt, Fabiola Martin, Martin Bland, William J Brackenbury

**Affiliations:** 1 Department of Health Sciences, University of York, York, UK; 2 Hull York Medical School, York, UK; 3 Department of Biology, University of York, York, UK

**Keywords:** PRIMARY CARE, STATISTICS & RESEARCH METHODS

## Abstract

**Introduction:**

Metastasis from solid tumours is associated with significant morbidity and mortality, and is the leading cause of cancer-related deaths. Voltage-gated sodium channels (VGSCs) are drug targets for the treatment of epilepsy. VGSCs are also present in cancer cells, where they regulate metastatic cell behaviours, including cellular movement and invasion. Treating cancer cells with the VGSC-inhibiting anticonvulsant phenytoin reduces cellular invasion and migration. Together, these suggest that VGSCs may be useful targets for inhibiting metastasis. The purpose of this study is to test the hypothesis that use of VGSC-inhibiting drugs will reduce metastasis, and therefore increase survival time in patients with cancer.

**Methods and analysis:**

A cohort study based on primary care data from the QResearch database will include patients with one of the three common tumours: breast, bowel and prostate. The primary outcome will be overall survival from the date of cancer diagnosis. Cox proportional hazards regression will be used to compare the survival of patients with cancer taking VGSC-inhibiting drugs (including anticonvulsants and class I antiarrhythmic agents) with patients with cancer not exposed to these drugs, adjusting for age and sex. Exposure to VGSC-inhibiting drugs will be defined as having at least one prescription for these drugs prior to cancer diagnosis. High and low exposure groups will be identified based on the length of use. A number of sensitivity and secondary analyses will be conducted.

**Ethics and dissemination:**

The protocol has been independently peer-reviewed and approved by the QResearch Scientific Board. The project has also been approved by the University of York Ethical Review Process. The results will be presented at international conferences and published in an open access peer-reviewed journal, in accordance with the Strengthening the Reporting of Observational Studies in Epidemiology (STROBE) criteria.

**Strengths and limitations of this study**

- Primary care research data.
- Large sample size and statistical power.
- Planned sensitivity analyses.
- Prescription-based study.
- No direct information on metastasis, estimation is via overall survival.
- Some variables of interest may be missing and/or poor quality in general practice data.

## Introduction

Bowel, breast and colon cancer are common cancers, which if diagnosed late have often already spread to secondary sites (metastasised). Metastasis is associated with significant morbidity and mortality. Metastasis is the leading cause of cancer-related deaths^1^ because a metastatic cancer is rarely amenable to cure, and interventions are largely limited to palliation.^2^ Therefore, there is an urgent need to identify and/or develop new metastasis prevention strategies.

The classical role of voltage-gated sodium channels (VGSCs) is to transmit action potentials in electrically excitable cells, for example, neurons and cardiomyocytes.^3^ VGSCs also regulate neuronal growth and migration.^4–7^ Related to these functions, VGSCs are clinical targets for a range of disorders, including epilepsy, cardiac arrhythmias, neuropathic pain and depression.^8^ The mode of action of a number of commonly prescribed antiepileptic drugs (anticonvulsants), including phenytoin, lamotrigine, carbamazepine and valproate, is to inhibit VGSCs.^9^ Similarly, the principal mode of action of class I antiarrhythmic drugs is to inhibit VGSCs.^10^

Recently, VGSCs have been identified in cells from a number of major cancers, including carcinomas of the breast, prostate and colon.^11^ ^12^ In these cells, VGSCs promote in vitro cellular behaviours that are associated with metastasis, including migration and invasion.^13–18^ Overexpression of the VGSC β1 subunit in breast cancer cells increases metastasis in mice.^19–21^ The VGSC-inhibiting anticonvulsant phenytoin significantly reduces migration and invasion of metastatic breast and prostate cancer cells in vitro.^22^ ^23^ Together, these data suggest that VGSCs may be useful targets for antimetastatic therapy, and that VGSC-inhibiting drugs may improve survival from certain cancers.^11^ ^24^ Although the effect of several anticonvulsants on risk of developing various cancers has been studied before (reviewed in ref. 25), the relationship between VGSC-inhibiting drugs and survival of patients with cancer has not been investigated.

The purpose of this study is to test the hypothesis that use of VGSC-inhibiting drugs will predict increased time to metastasis and thus improved survival time in patients with cancer. The objectives are to investigate:

- The relationship between use of all VGSC-inhibiting (anticonvulsant and class I antiarrhythmic) drugs and overall survival of patients with cancer. We will focus on carcinomas of the breast, colon and prostate because they are the most common and VGSC expression has been extensively studied in these tumours.^11^ ^13–17^ ^26–29^
- The relationship between use of all VGSC-inhibiting drugs and cancer-specific survival.
- The relationship between individual VGSC-inhibiting drugs and overall survival.

There are no systematic reviews exploring this area and we are addressing this gap by conducting a review concurrent to this study PROSPERO registration number CRD42014013574.

## Methods and analysis

### Data source and sample selection

This study will use general practice (GP) data accessed from QResearch (http://www.qresearch.org), a large consolidated database derived from the anonymised health records of over 13 million patients from 753 GPS (representing around 7% of the UK practices). QResearch data are collected from the EMIS GP computer system and have been validated using other sources and shown to yield similar results to other databases, for example, the Clinical Practice Research Datalink (CPRD).^30^ ^31^ QResearch has been used previously to study associations between cancer and prescription information.^30^

An open cohort of 100 000 patients (aged 30 years or older) with a diagnosis of breast, colorectal or prostate cancer will be identified who were registered with a QResearch practice during the study period between 1 January 1998 and 31 December 2013. This will include all those patients with cancer in the database who have a prescription of one of the index drugs recorded before their date of cancer diagnosis (table 1).^32^ The remaining patients will be randomly selected controls. Time from date of diagnosis to death will be investigated and data will be right-censored in patients who are still alive at the end of the study period. Cancer diagnoses will be based on Read code information (available online at clinicalcodes.org/medcodes/article/17/).

**Table 1:** Voltage-gated Na^+^ channel-inhibiting drugs

Drug/derivative	Alternative names	Classification	British National Formulary section^32^
Carbamazapine, eslicarbazepine, oxcarbazepine	Arbil, carbagen SR, epimaz, inovelon, tegretol, teril, timonil, trileptal, zebinix	Anticonvulsant	4.2.3, 4.7.3, 4.8.1
Disopyramide	Dirythmin, isomide, rythmodan	Class Ia antiarrhythmic	2.3.2
Flecainide	Tambocor	Class Ic antiarrhythmic	2.3.2
Lacosamide	Vimpat	Anticonvulsant	4.8.1
Lamotrigine	Lamictal	Anticonvulsant	4.8.1
Lidocaine	Lignocaine, xylocard	Class Ib antiarrhythmic	2.3.2, 15.2
Mexiletine	Mexitil	Class Ib antiarrhythmic	2.3.2
Moracizine	Ethmozine	Class Ic antiarrhythmic	–
Phenytoin, fosphenytoin	Epanutin, pentran	Anticonvulsant, class Ib antiarrhythmic	4.7.3, 4.8.1, 4.8.2
Procainamide	Pronestyl	Class Ia antiarrhythmic	2.3.2
Propafenone	Arythmol	Class Ic antiarrhythmic	2.3.2
Quinidine	Kiditard	Class Ia antiarrhythmic	–
Ranolazine	Ranexa	Antianginal	2.6.3
Riluzole	Rilutek	Treatment for amyotrophic lateral sclerosis	4.9.3
Tocainide	Tonocard	Class Ib antiarrhythmic	–
Topiramate	Topamax	Anticonvulsant	4.7.4, 4.8.1
Valproic acid, sodium valproate	Convulex, depakote, epilim, epival, episenta, orlept	Anticonvulsant	4.2.3, 4.7.4, 4.8.1

### Exclusions

Temporary residents and patients registered with QResearch within 12 months of data extraction will be excluded. Cases without diagnosis of one of the three index cancers (breast, colorectal or prostate cancer) will be excluded. Patients with anomalous, incorrect or infeasible dates will be excluded, for example, dates of cancer diagnoses recorded before birth or after death. We shall assume that dates of birth and death are correct. Any patient with a date of diagnosis that indicates they were younger than 25 at the time of diagnosis will be excluded as it is unlikely a person of that age would get one of these three index cancers.

### Exposure

A participant will be considered as exposed if they have had at least one prescription for one of the index drugs. Assuming continuous treatment use between prescriptions, we will identify two exposure groups: a *low* exposure group (less than 6 months’ worth of prescriptions) and a *moderate to high* exposure group (6 months or more prescriptions). The exposed groups, separately and in combination, will be compared with the control group (cases without any prescription for one of the index drugs). Patients with one prescription for a drug that would have been used as a local anaesthetic, for example, lidocaine, will be excluded.

### Outcome measures

Metastasis is estimated to be responsible for 90% of deaths from solid tumours.^33^ However, metastasis itself is not reliably recorded in GP data and so the primary outcome measure will be overall survival following cancer diagnosis as a proxy for metastasis. Secondary outcome measures will be cancer-specific survival for each index type of cancer and overall survival across each drug, numbers permitting.

### Confounding factors

Data on the following confounders will be requested: age, gender, alcohol consumption, smoking status, body mass index (BMI) and ethnicity. Data on alcohol, smoking and BMI are routinely collected and as such a single patient may have multiple recorded observations for these variables assessed over time. We will consider the observations measured at the closest date before the date of cancer diagnosis, based on appropriate Read codes (available online at clinicalcodes.org/medcodes/article/17/). The patients will be categorised as follows:

- Alcohol consumption^34^ categorised as non-drinker/trivial drinker (<1 unit/day), light drinker (1–2 units/day), moderate–very heavy drinker (3+units/day) and not recorded/known.
- Smoking status^35^ categorised as ex-smoker, smoker, non-smoker and not recorded/known.
- BMI^36^ categorised as underweight (<18.5), normal range (18.5–25), overweight (25–30), obese (30+) and not recorded/known.
- Ethnicity^37^ categorised according to the groupings used in the 2011 UK census: white, mixed/multiple ethnic groups, Asian/Asian British, black/African/Caribbean/black British, other ethnic group. We shall also include a ‘Not recorded/known’ category.

### Sample size calculation

Up to 100 000 eligible cases will be used, which is the maximum sample size that will be released by QResearch. At breast cancer diagnosis, approximately 6% of patients present with metastatic lesions, with bone being the most common site.^38^ Of patients presenting without bone metastasis at diagnosis, 3.6% subsequently develop metastases.^39^ ^40^ The majority (90%) of metastases will lead to death.^33^ Pharmacological blockade of VGSCs inhibits invasion of breast, colorectal and prostate cancer cells in vitro by 25–50%.^13^ ^15^ ^22^ ^23^ Therefore, assuming 3.6% of cancer diagnoses lead to a metastasis and most of these to death, with standard significance level α=5% and power=90%, we would require 4248 in the exposed group to detect a fall of 25% in the metastasis (or death) rate and 928 to detect a fall of 50%. This is based on 20 comparison patients per exposed patient, but this ratio is not critical. If we include 6% with a metastasis present at initial diagnosis, these numbers fall to 1503 and 330.

The prevalence of epilepsy is estimated to be 1%.^41^ Together, the most commonly used VGSC-inhibiting anticonvulsants, phenytoin, lamotrigine, carbamazepine and valproate, account for >82% of all antiepileptic drug use.^42^ By contrast, class I antiarrhythmic drug use has been considerably less common: <5% in patients with cardiac arrhythmia.^43^ Thus, using these data as a guide, we might reasonably anticipate that around 0.8% of patients with cancer would be using one of these VGSC-inhibiting drugs. To meet our largest target sample size, 4248, we would therefore be looking for a sample that contained 530 000 people with a diagnosis of one of the target cancers. To meet the lower target of 928, we would require 116 000 diagnoses. Given that we are studying deaths rather than metastases per se, we will be unable to distinguish between metastases present at diagnosis and detected subsequently. Therefore, if we include 6% assumed to have a metastasis present at initial diagnosis, we would require 187 875 and 41 250 diagnoses to detect falls in metastasis of 25% and 50%, respectively.

According to Cancer Research UK,^44^ the lifetime risk in 2010 for the four major cancer sites was almost 13% (female breast), 6% (female lung), 8% (male lung), 6% (female bowel including anus), 7% (male bowel including anus) and 13% (prostate). Hence for our chosen sites, we expect approximately 21% of women and 20% of men to experience a positive diagnosis at some time. We will not have lifetime data for many in the database, but we might anticipate that 10% of a database sample would have a history of one of these sites. Thus the QResearch database of 13 million people is large enough to achieve our largest sample target.

### Statistical analysis

Analysis will be conducted in Stata V.13, using two-sided significance at the 5% level. For each Cox model, only the patients with complete data for each of the covariates controlled for in the model will be included in the analysis.

#### Descriptive summaries

The characteristics of the comparison groups will be described using summary statistics. Categorical data will be presented as frequency and percentage, and continuous variables will be summarised using descriptive statistics (mean, SD, median, 1st and 3rd quartiles, minimum and maximum). The flow of patients in the QResearch database will be presented in a diagram.

#### Primary analysis

The primary analysis will compare the combined exposure group with the control group. For each group, the distribution of time from diagnosis of cancer to death will be described using Kaplan-Meier survival estimates. Kaplan-Meier survival curves will be presented for the two groups. The statistical equivalence of the two curves will be tested using the log-rank test. Right censoring will occur if the patient is still alive at the end of the study period (31 December 2013). Median time to death, with a 95% CI will be presented. If the estimated survivor function is greater than 0.5 throughout the study it will not be possible to estimate the median survival time and other percentiles’ survival values (ie, 90%, 80%, 75%, as appropriate) will be presented.

We will compare the survival of exposed cases with control cases from the time of diagnosis of one of the three index cancers using a Cox proportional hazards regression model. The end point will be all-cause mortality. We will adjust the Cox model for type of cancer (breast, bowel or prostate), gender and age at diagnosis. Age will be included with a linear as well as a quadratic term (age+age^2^). We will assume that all included patients are receiving the most appropriate standard treatment for their disease, so we will not adjust for cancer-treating drug intake. HRs will be presented with p values and 95% CIs.

Cox regression assumes that the proportional hazards model applies. To assess this, we shall plot −log(−log(S(t))) against log(time), where S(t) is the survivor function at time t. The curves for the two groups should be parallel. We will also consider a χ^2^ test of the Schoenfeld residuals to assess the null hypothesis of no relationship between the hazards in each group. If the assumptions are not met, we shall try to investigate why this is.

#### Sensitivity analysis

We will repeat the primary analysis, but adjust the Cox model, in turn, for confounding variables: ethnicity, BMI, smoking and alcohol consumption.

#### Secondary analyses

Each of the following secondary end points will be analysed like the primary outcome (unless indicated) with identical censoring strategy.

- If cancer type proves to be a significant predictor in the primary model then we will consider cancer-specific survival;
- Survival of low exposure group compared with control group;
- Survival of high exposure group compared with control group;
- Survival of combined exposure group and control group with outcome of time to death from first diagnosis of any cancer, since some patients may have a diagnosis of another cancer before one of breast, bowel or prostate (a category for ‘Other’ will be included in the covariate for type of cancer);
- Survival of patients dependent on the main drug class that they are exposed to (numbers permitting).

## Ethics and dissemination

This protocol has been independently peer-reviewed by the QResearch Scientific Board. Only the authors will have access to the data during the study, in order to guarantee confidentiality of patient information. An article detailing the results of the study will be submitted for publication in an international peer-reviewed journal, in accordance with the Strengthening the Reporting of Observational Studies in Epidemiology (STROBE) criteria.^45^ The full statistical analysis will be available from the authors after publication of the results.

## Supplementary Material

Author's manuscript

Reviewer comments
