# Supplementary material for: Exposure to sodium channel-inhibiting drugs and cancer survival: protocol for a cohort study using the QResearch primary care database
Source: BMJ Open. 2014 Nov 14;4(11):e006604. doi: 10.1136/bmjopen-2014-006604 (PMC4244419; doi:10.1136/bmjopen-2014-006604)
Supplement: Author's manuscript [file bmjopen-2014-006604.draft_revisions.pdf]

Exposure to sodium channel-inhibiting drugs and cancer survival: protocol for a cohort study using the QResearch primary care database

Journal:	*BMJ Open*
Manuscript ID:	bmjopen-2014-006604
Article Type:	Protocol
Date Submitted by the Author:	11-Sep-2014
Complete List of Authors:	Fairhurst, Caroline; University of York, Health Sciences<br>Watt, Ian; University of York, Health Sciences<br>Martin, Fabiola; Hull York Medical School,<br>Bland, Martin; University of York, Health Sciences<br>Brackenbury, William; University of York, Biology
**Primary Subject Heading**:	Oncology
Secondary Subject Heading:	Epidemiology, Pharmacology and therapeutics
Keywords:	PRIMARY CARE, Epilepsy < NEUROLOGY, Breast tumours < ONCOLOGY, Epidemiology < ONCOLOGY, STATISTICS & RESEARCH METHODS, Clinical trials < THERAPEUTICS

SCHOLARONE™
Manuscripts

Exposure to sodium channel-inhibiting drugs and cancer survival: protocol for a cohort study using the QResearch primary care database

Caroline Fairhurst[1], Ian Watt[1,2], Fabiola Martin[2,3], Martin Bland[1], and William J. Brackenbury[3*]

[1]Department of Health Sciences, University of York, York, UK

[2]Hull York Medical School, York, UK

[3]Department of Biology, University of York, York, UK

*Corresponding author:

William J. Brackenbury

Department of Biology, University of York, Heslington, York, YO10 5DD, UK

E-mail: william.brackenbury@york.ac.uk

Telephone: +44 (0) 1904 328 284

Keywords: Anticonvulsants / Breast neoplasms / Colonic neoplasms / Prostatic neoplasms / Sodium channels

Word count, excluding title page, abstract, references, figures and tables: 2327.

For peer review only

ABSTRACT

Introduction: Metastasis from solid tumours is associated with significant morbidity and mortality, and is the leading cause of cancer-related deaths. Voltage-gated sodium channels (VGSCs) are drug targets for the treatment of epilepsy. VGSCs are also present in cancer cells, where they regulate metastatic cell behaviours, including cellular movement and invasion. Treating cancer cells with the VGSC-inhibiting anticonvulsant phenytoin reduces cellular invasion and migration. Together, these suggest that VGSCs may be useful targets for inhibiting metastasis. The purpose of this study is to test the hypothesis that use of VGSC-inhibiting drugs will reduce metastasis, and therefore increase survival time in cancer patients.

Methods and analysis: A cohort study based on primary care data from the QResearch database will include patients with one of three common tumours: breast, bowel, and prostate. The primary outcome will be overall survival from date of cancer diagnosis. Cox proportional hazards regression will be used to compare the survival of cancer patients taking VGSC-inhibiting drugs (including anticonvulsants and Class I antiarrhythmic agents) with cancer patients not exposed to these drugs, adjusting for age and sex. Exposure to VGSC-inhibiting drugs will be defined as having at least one prescription for these drugs prior to cancer diagnosis. High and low exposure groups will be identified based on length of use. A number of sensitivity and secondary analyses will be conducted.

Ethics and dissemination: The protocol has been independently peer-reviewed and approved by the QResearch Scientific Board. The project has also been approved by the University of York Ethical Review Process. The results will be presented at international conferences and published in an open access peer-reviewed journal, in accordance with the STROBE criteria.

For peer review only

STRENGTHS AND LIMITATIONS OF THIS STUDY

- Primary care research data
- Large sample size and statistical power
- Planned sensitivity analyses
- Prescription-based study
- No direct information on metastasis, estimation is *via* overall survival
- Some variables of interest may be missing and/or poor quality in GP data

INTRODUCTION

Bowel, breast and colon cancer are common cancers, which if diagnosed late have often already spread to secondary sites (metastasised). Metastasis is associated with significant morbidity and mortality. Metastasis is the leading cause of cancer-related deaths[1] because a metastatic cancer is rarely amenable to cure, and interventions are largely limited to palliation.[2] Therefore, there is an urgent need to identify and/or develop new metastasis prevention strategies.

The classical role of voltage-gated sodium channels (VGSCs) is to transmit action potentials in electrically excitable cells, e.g. neurons and cardiomyocytes.[3] VGSCs also regulate neuronal growth and migration.[4-7] Related to these functions, VGSCs are clinical targets for a range of disorders, including epilepsy, cardiac arrhythmias, neuropathic pain and depression.[8] The mode of action of a number of commonly prescribed antiepileptic drugs (anticonvulsants), including phenytoin, lamotrigine, carbamazepine and valproate, is to inhibit VGSCs.[9] Similarly, the principal mode of action of Class I antiarrhythmic drugs is to inhibit VGSCs.[10]

For peer review only

More recently, VGSCs have been identified in cells from a number of major cancers, including carcinomas of the breast, prostate and colon.[11 12] In these cells, VGSCs promote *in vitro* cellular behaviours that are associated with metastasis, including migration and invasion.[13-18] Overexpression of the VGSC β1 subunit in breast cancer cells increases metastasis in mice.[19-21] The VGSC-inhibiting anticonvulsant phenytoin significantly reduces migration and invasion of metastatic breast and prostate cancer cells *in vitro*[22 23] and has been shown to reduce metastasis in a mouse model of breast cancer (M. Nelson, M. Yang, A. A. Dowle, J. R. Thomas and W. J. Brackenbury, manuscript under review, 2014). Together, these data suggest that VGSCs may be useful targets for anti-metastatic therapy, and that VGSC-inhibiting drugs may improve survival from certain cancers.[11 24] Although the effect of several anticonvulsants on risk of developing various cancers has been studied before (reviewed in[25]), the relationship between VGSC-inhibiting drugs and survival of cancer patients has not been investigated.

The purpose of this study is to test the hypothesis that use of VGSC-inhibiting drugs will predict increased time to metastasis and thus improved survival time in cancer patients. The objectives are to investigate:

- The relationship between use of all VGSC-inhibiting (anticonvulsant and Class I antiarrhythmic) drugs and overall survival of cancer patients. We will focus on carcinomas of the breast, colon and prostate because they are the most common and VGSC expression has been extensively studied in these tumours.[11 13-17 26-30]
- The relationship between use of all VGSC-inhibiting drugs and cancer specific survival.

For peer review only

- The relationship between individual VGSC-inhibiting drugs and overall survival.

There are no systematic reviews exploring this area and we are addressing this gap by conducting a review concurrent to this study [PROSPERO registration number CRD42014013574].

METHODS AND ANALYSIS

Data source and sample selection

This study will use general practice data accessed from QResearch (http://www.qresearch.org), a large consolidated database derived from the anonymised health records of over 13 million patients from 753 general practices (representing around 7% of UK practices). QResearch data are collected from the EMIS GP computer system and have been validated using other sources and shown to yield similar results to other databases, e.g. the Clinical Practice Research Datalink (CPRD).[31 32] QResearch has been used previously to study associations between cancer and prescription information.[31]

An open cohort of 100,000 patients (aged 30 years or older) with a diagnosis of breast, colorectal or prostate cancer will be identified who were registered with a QResearch practice during the study period between 1st January 1998 and 31st December 2013. This will include all those cancer patients in the database who have a prescription of one of the index drugs recorded before their date of cancer diagnosis (Table 1).[33] The remaining patients will be randomly selected controls. Time from date of diagnosis to death will be investigated and data will be right-censored in patients who are still alive at the end of the study period. Cancer

For peer review only - http://bmjopen.bmj.com/site/about/guidelines.xhtml

diagnoses will be based on Read code information (see online supplementary appendix).

Table 1. Voltage-gated Na^+ channel-inhibiting drugs

Drug/derivative	Alternative names	Classification	British National Formulary (BNF) section[33]
Carbamazapine, eslicarbazepine, oxcarbazepine	Arbil, Carbagen SR, Epimaz, Inovelon, Tegretol, Teril, Timonil, Trileptal, Zebinix	Anticonvulsant	4.2.3, 4.7.3, 4.8.1
Disopyramide	Dirythmin, Isomide, Rythmodan	Class Ia antiarrhythmic	2.3.2
Flecainide	Tambocor	Class Ic antiarrhythmic	2.3.2
Lacosamide	Vimpat	Anticonvulsant	4.8.1
Lamotrigine	Lamictal	Anticonvulsant	4.8.1
Lidocaine	Lignocaine, Xylocard	Class Ib antiarrhythmic	2.3.2, 15.2
Mexiletine	Mexitil	Class Ib antiarrhythmic	2.3.2
Moracizine	Ethmozine	Class Ic antiarrhythmic	-
Phenytoin, fosphenytoin	Epanutin, Pentran	Anticonvulsant, Class Ib antiarrhythmic	4.7.3, 4.8.1, 4.8.2

For peer review only

Procainamide	Pronestyl	Class Ia antiarrhythmic	2.3.2
Propafenone	Arythmol	Class Ic antiarrhythmic	2.3.2
Quinidine	Kiditard	Class Ia antiarrhythmic	-
Ranolazine	Ranexa	Antianginal	2.6.3
Riluzole	Rilutek	Treatment for amyotrophic lateral sclerosis	4.9.3
Tocainide	Tonocard	Class Ib antiarrhythmic	-
Topiramate	Topamax	Anticonvulsant	4.7.4, 4.8.1
Valproic acid, sodium valproate	Convulex, Depakote, Epilim, Epival, Episenta, Orlept	Anticonvulsant	4.2.3, 4.7.4, 4.8.1

Exclusions

Temporary residents and patients registered with QResearch within 12 months of data extraction will be excluded. Cases without diagnosis of one of the three index cancers (breast, colorectal, or prostate cancer) will be excluded. Patients with anomalous, incorrect or infeasible dates will be excluded, e.g., dates of cancer diagnoses recorded before birth or after death. We shall assume that dates of birth and death are correct. Any patient with a date of diagnosis that indicates they were

For peer review only

younger than 25 at the time of diagnosis will be excluded as it is unlikely a person of that age would get one of these three cancers.

Exposure

A participant will be considered as exposed if they have had at least one prescription for one of the index drugs. Assuming continuous treatment use between prescriptions, we will identify two exposure groups: a *low* exposure group (less than 6 months worth of prescriptions) and a *moderate to high* exposure group (six months or more prescriptions). The exposed groups, separately and in combination, will be compared with the control group (cases without any prescription for one of the index drugs). Patients with one prescription for a drug that would have been used as a local anaesthetic, e.g. lidocaine, will be excluded.

Outcome measures

Metastasis is estimated to be responsible for 90% of deaths from solid tumours.[34] However, metastasis itself is not reliably recorded in GP data and so the primary outcome measure will be overall survival following cancer diagnosis as a proxy for metastasis. Secondary outcome measures will be cancer-specific survival for each index type of cancer and overall survival across each drug, numbers permitting.

Confounding factors

Data on the following confounders will be requested: age, gender, alcohol consumption, smoking status, body mass index (BMI) and ethnicity. Data on alcohol, smoking and BMI are routinely collected and as such a single patient may have multiple recorded observations for these variables assessed over time. We will

For peer review only

consider the observations measured at the closest date before the date of cancer diagnosis, based on appropriate Read codes (detailed in online supplementary appendix). The patients will be categorised as follows:

- Alcohol consumption[35] categorised as: Non/trivial drinker (<1unit/day), Light drinker (1-2units/day), Moderate-very heavy drinker(3+units/day) and Not recorded/known.
- Smoking status[36] categorised as: Ex-smoker, Smoker, Non-smoker, and Not recorded/known.
- BMI,[37] categorised as Underweight (<18.5), Normal range [18.5-25), Overweight [25, 30), Obese (30+) and Not recorded/known.
- Ethnicity[38] categorised according to the groupings used in the 2011 UK census: White; Mixed/Multiple ethnic groups; Asian/Asian British; Black/African/Caribbean/Black British; Other Ethnic group. We shall also include a 'Not recorded/known' category.

Sample size calculation

Up to 100,000 eligible cases will be used, which is the maximum sample size that will be released by QResearch. At breast cancer diagnosis, approximately 6% of patients present with metastatic lesions, with bone being the most common site.[39] Of patients presenting without bone metastasis at diagnosis, 3.6% subsequently develop metastases.[40 41] The majority (90%) of metastases will lead to death.[34] Pharmacological blockade of VGSCs inhibits invasion of breast, colorectal and prostate cancer cells *in vitro* by 25-50%.[13 15 22 23] Therefore, assuming 3.6% of cancer diagnoses lead to a metastasis and most of these to death, with standard significance level alpha = 5% and power = 90%, we would require 4248 in the exposed group to detect a fall of 25% in the metastasis (or death) rate and 928 to

For peer review only - http://bmjopen.bmj.com/site/about/guidelines.xhtml

detect a fall of 50%. This is based on 20 comparison patients per exposed patient, but this ratio is not critical. If we include 6% with a metastasis present at initial diagnosis, these numbers fall to 1503 and 330.

The prevalence of epilepsy is estimated to be 1%.[42] Together, the most commonly used VGSC-inhibiting anticonvulsants, phenytoin, lamotrigine, carbamazepine and valproate, account for >82% of all antiepileptic drug use.[43] By contrast, Class I antiarrhythmic drug use has been considerably less common: <5% in patients with cardiac arrhythmia.[44] Thus, using these data as a guide, we might reasonably anticipate that around 0.8% of cancer patients would be using one of these VGSC-inhibiting drugs. To meet our largest target sample size, 4248, we would therefore be looking for a sample that contained 530,000 people with a diagnosis of one of the target cancers. To meet the lower target of 928, we would require 116,000 diagnoses. Given that we are studying deaths rather than metastases *per se*, we will be unable to distinguish between metastases present at diagnosis and detected subsequently. Therefore, if we include 6% assumed to have a metastasis present at initial diagnosis, we would require 187,875 and 41,250 diagnoses to detect falls in metastasis of 25% and 50% respectively.

According to Cancer Research UK,[45] the lifetime risk in 2010 for the four major cancer sites was almost 13% (female breast), 6% (female lung), 8% (male lung), 6% (female bowel including anus), 7% (male bowel including anus) and 13% (prostate). Hence for our chosen sites, we expect approximately 21% of women and 20% of men to experience a positive diagnosis at some time. We will not have lifetime data for many in the database, but we might anticipate that 10% of a database sample would have a history of one of these sites. Thus the Qresearch database of 13 million people is large enough to achieve our largest sample target.

For peer review only - http://bmjopen.bmj.com/site/about/guidelines.xhtml

Statistical analysis

Analysis will be conducted in Stata v13, using two-sided significance at the 5% level. For each Cox model, only the patients with complete data for each of the covariates controlled for in the model will be included in the analysis.

Descriptive summaries

The characteristics of the comparison groups will be described using summary statistics. Categorical data will be presented as frequency and percentage, and continuous variables will be summarised using descriptive statistics (mean, standard deviation, median, 1st and 3rd quartiles, minimum and maximum). The flow of patients in the QResearch database will be presented in a diagram.

Primary analysis

The primary analysis will compare the combined exposure group to the control group. For each group, the distribution of time from diagnosis of cancer to death will be described using Kaplan-Meier survival estimates. Kaplan-Meier survival curves will be presented for the two groups. The statistical equivalence of the two curves will be tested using the log-rank test. Right censoring will occur if the patient is still alive at the end of the study period (31st December 2013). Median time to death, with a 95% confidence interval (CI) will be presented. If the estimated survivor function is greater than 0.5 throughout the study it will not be possible to estimate the median survival time and other percentiles survival values (i.e. 90%, 80%, 75%, as appropriate) will be presented.

For peer review only

We will compare the survival of exposed cases with control cases from time of diagnosis of one of the three index cancers using a Cox Proportional Hazards regression model. The endpoint will be all cause mortality. We will adjust the Cox model for type of cancer (breast, bowel or prostate), gender and age at diagnosis. Age will be included with both a linear and quadratic term (age + age^2). We will assume that all included patients are receiving the most appropriate standard treatment for their disease, so we will not adjust for cancer-treating drug intake. Hazard ratios will be presented with p-values and 95% confidence intervals.

Cox regression assumes that the proportional hazards model applies. To assess this, we shall plot –log(-log(S(t))) against log(time), where S(t) is the survivor function at time *t*. The curves for the two groups should be parallel. We will also consider a chi-squared test of the Schoenfeld residuals to assess the null hypothesis of no relationship between the hazards in each group. If the assumptions are not met, we shall try to investigate why this is.

Sensitivity analysis

We will repeat the primary analysis, but adjust the Cox model, in turn, for confounding variables: ethnicity, body mass index, smoking and alcohol consumption.

Secondary analyses

Each of the following secondary end points will be analysed like the primary outcome (unless indicated) with identical censoring strategy:

- If cancer type proves to be a significant predictor in the primary model then we will consider cancer specific survival

For peer review only

- Survival of Low exposure group compared with control group
- Survival of High exposure group compared with control group
- Survival of combined exposure group and control group with outcome of time to death from first diagnosis of any cancer, since some patients may have a diagnosis of another cancer before one of breast, bowel or prostate (a category for ‘Other’ will be included in the covariate for type of cancer)
- Survival of patients dependent on the main drug class that they are exposed to (numbers permitting).

ETHICS AND DISSEMINATION

This protocol has been independently peer-reviewed by the QResearch Scientific Board. It has also been approved by the University of York Ethical Review Process. Only the authors will have access to the data during the study, in order to guarantee confidentiality of patient information. An article detailing the results of the study will be submitted for publication in an international peer-reviewed journal, in accordance with the Strengthening the Reporting of Observational Studies in Epidemiology (STROBE) criteria.[46] The full protocol and statistical analysis will be available from the authors after publication of the results.

AUTHORS’ CONTRIBUTIONS

WB had the original idea for this study. CF and WB wrote the draft of the manuscript. IW, FM and MB contributed to the development of the idea, the study design, and revised the manuscript. All authors approved the final submitted version of the manuscript.

For peer review only

FUNDING STATEMENT

This work was supported by the Medical Research Council [Fellowship G1000508] and the Wellcome Trust [ref: 097829] through the Centre for Chronic Diseases and Disorders (C2D2) at the University of York.

COMPETING INTERESTS STATEMENT

The authors declare that they have no competing interests.

REFERENCES

1. Rugo HS. The importance of distant metastases in hormone-sensitive breast cancer. Breast 2008;**17 Suppl 1**:S3-8 doi: 10.1016/S0960-9776(08)70002-Xpublished Online First: Epub Date]|.
2. Suva LJ, Griffin RJ, Makhoul I. Mechanisms of bone metastases of breast cancer. Endocr Relat Cancer 2009;**16**(3):703-13 doi: 10.1677/ERC-09-0012published Online First: Epub Date]|.
3. Hille B. *Ionic channels of excitable membranes*. 2nd ed. Sunderland (Massachusetts): Sinauer Associates Inc., 1992.
4. Brackenbury WJ, Calhoun JD, Chen C, et al. Functional reciprocity between Na+ channel Nav1.6 and β1 subunits in the coordinated regulation of excitability and neurite outgrowth. Proc Natl Acad Sci U S A 2010;**107**(5):2283-88
5. Brackenbury WJ, Yuan Y, O'Malley HA, Parent JM, Isom LL. Abnormal neuronal patterning occurs during early postnatal brain development of Scn1b-null mice and precedes hyperexcitability. Proc Natl Acad Sci U S A 2013;**110**(3):1089-94 doi: 10.1073/pnas.1208767110published Online First: Epub Date]|.
6. Catterall WA. From ionic currents to molecular mechanisms: the structure and function of voltage-gated sodium channels. Neuron 2000;**26**(1):13-25
7. Brackenbury WJ, Davis TH, Chen C, et al. Voltage-gated Na^+ channel β1 subunit-mediated neurite outgrowth requires fyn kinase and contributes to central nervous system development in vivo. J Neurosci 2008;**28**(12):3246-56
8. Clare JJ, Tate SN, Nobbs M, Romanos MA. Voltage-gated sodium channels as therapeutic targets. Drug Discov Today 2000;**5**(11):506-20
9. Mantegazza M, Curia G, Biagini G, Ragsdale DS, Avoli M. Voltage-gated sodium channels as therapeutic targets in epilepsy and other neurological disorders. Lancet Neurol 2010;**9**(4):413-24 doi: 10.1016/S1474-4422(10)70059-4published Online First: Epub Date]|.
10. Vaughan Williams EM. The relevance of cellular to clinical electrophysiology in classifying antiarrhythmic actions. J Cardiovasc Pharmacol 1992;**20 Suppl 2**:S1-7

For peer review only - http://bmjopen.bmj.com/site/about/guidelines.xhtml

11. Brackenbury WJ. Voltage-gated sodium channels and metastatic disease. Channels (Austin) 2012;**6**(5):352-61 doi: 10.4161/chan.21910published Online First: Epub Date]|.
12. Brackenbury WJ, Djamgoz MB, Isom LL. An emerging role for voltage-gated Na+ channels in cellular migration: regulation of central nervous system development and potentiation of invasive cancers. Neuroscientist 2008;**14**(6):571-83 doi: 10.1177/1073858408320293published Online First: Epub Date]|.
13. House CD, Vaske CJ, Schwartz A, et al. Voltage-gated Na+ channel SCN5A is a key regulator of a gene transcriptional network that controls colon cancer invasion. Cancer Res 2010;**70**(17):6957-67 doi: 10.1158/0008-5472.CAN-10-1169published Online First: Epub Date]|.
14. Grimes JA, Fraser SP, Stephens GJ, et al. Differential expression of voltage-activated Na^+ currents in two prostatic tumour cell lines: contribution to invasiveness in vitro. FEBS Lett 1995;**369**(2-3):290-4
15. Fraser SP, Diss JK, Chioni AM, et al. Voltage-gated sodium channel expression and potentiation of human breast cancer metastasis. Clin Cancer Res 2005;**11**(15):5381-89 doi: 10.1158/1078-0432.CCR-05-0327published Online First: Epub Date]|.
16. Brackenbury WJ, Chioni AM, Diss JK, Djamgoz MB. The neonatal splice variant of Nav1.5 potentiates in vitro metastatic behaviour of MDA-MB-231 human breast cancer cells. Breast Cancer Res. Treat. 2007;**101**(2):149-60
17. Brackenbury WJ, Djamgoz MB. Activity-dependent regulation of voltage-gated Na^+ channel expression in Mat-LyLu rat prostate cancer cell line. J Physiol 2006;**573**(Pt 2):343-56 doi: 10.1113/jphysiol.2006.106906published Online First: Epub Date]|.
18. Brackenbury WJ, Djamgoz MB. Nerve growth factor enhances voltage-gated Na+ channel activity and Transwell migration in Mat-LyLu rat prostate cancer cell line. J Cell Physiol 2007;**210**(3):602-8 doi: 10.1002/jcp.20846published Online First: Epub Date]|.
19. Nelson M, Millican-Slater R, Forrest LC, Brackenbury WJ. The sodium channel beta1 subunit mediates outgrowth of neurite-like processes on breast cancer cells and promotes tumour growth and metastasis. Int J Cancer 2014;**135**(10):2338-51 doi: 10.1002/ijc.28890published Online First: Epub Date]|.
20. Brackenbury WJ, Isom LL. Na Channel beta Subunits: Overachievers of the Ion Channel Family. Frontiers in pharmacology 2011;**2**:53 doi: 10.3389/fphar.2011.00053published Online First: Epub Date]|.
21. Chioni AM, Brackenbury WJ, Calhoun JD, Isom LL, Djamgoz MB. A novel adhesion molecule in human breast cancer cells: voltage-gated Na+ channel beta1 subunit. Int J Biochem Cell Biol 2009;**41**(5):1216-27 doi: 10.1016/j.biocel.2008.11.001published Online First: Epub Date]|.
22. Fraser SP, Salvador V, Manning EA, et al. Contribution of functional voltage-gated Na^+ channel expression to cell behaviors involved in the metastatic cascade in rat prostate cancer: I. lateral motility. J Cell Physiol 2003;**195**(3):479-87
23. Yang M, Kozminski DJ, Wold LA, et al. Therapeutic potential for phenytoin: targeting Na(v)1.5 sodium channels to reduce migration and invasion in metastatic breast cancer. Breast Cancer Res Treat 2012;**134**(2):603-15 doi: 10.1007/s10549-012-2102-9published Online First: Epub Date]|.
24. Brackenbury WJ, Isom LL. Voltage-gated Na+ channels: potential for beta subunits as therapeutic targets. Expert Opin Ther Targets 2008;**12**(9):1191-203 doi: 10.1517/14728222.12.9.1191published Online First: Epub Date]|.

For peer review only

25. Singh G, Driever PH, Sander JW. Cancer risk in people with epilepsy: the role of antiepileptic drugs. Brain 2005;**128**(Pt 1):7-17 doi: 10.1093/brain/awh363published Online First: Epub Date]|.
26. Brisson L, Driffort V, Benoist L, et al. NaV1.5 Na(+) channels allosterically regulate the NHE-1 exchanger and promote the activity of breast cancer cell invadopodia. J Cell Sci 2013;**126**(Pt 21):4835-42 doi: 10.1242/jcs.123901published Online First: Epub Date]|.
27. Ding L, Ellis MJ, Li S, et al. Genome remodelling in a basal-like breast cancer metastasis and xenograft. Nature 2010;**464**(7291):999-1005 doi: 10.1038/nature08989published Online First: Epub Date]|.
28. Grimes JA, Djamgoz MB. Electrophysiological characterization of voltage-gated Na(+) current expressed in the highly metastatic Mat-LyLu cell line of rat prostate cancer. J Cell Physiol 1998;**175**(1):50-8
29. Gillet L, Roger S, Besson P, et al. Voltage-gated Sodium Channel Activity Promotes Cysteine Cathepsin-dependent Invasiveness and Colony Growth of Human Cancer Cells. J Biol Chem 2009;**284**(13):8680-91 doi: M806891200 [pii] 10.1074/jbc.M806891200published Online First: Epub Date]|.
30. Ding Y, Brackenbury WJ, Onganer PU, et al. Epidermal growth factor upregulates motility of Mat-LyLu rat prostate cancer cells partially via voltage-gated Na^+ channel activity. J Cell Physiol 2008;**215**(1):77-81 doi: 10.1002/jcp.21289published Online First: Epub Date]|.
31. Vinogradova Y, Coupland C, Hippisley-Cox J. Exposure to bisphosphonates and risk of gastrointestinal cancers: series of nested case-control studies with QResearch and CPRD data. BMJ (Clinical research ed.) 2013;**346**:f114 doi: 10.1136/bmj.f114published Online First: Epub Date]|.
32. Reeves D, Springate DA, Ashcroft DM, et al. Can analyses of electronic patient records be independently and externally validated? The effect of statins on the mortality of patients with ischaemic heart disease: a cohort study with nested case-control analysis. BMJ open 2014;**4**(4):e004952 doi: 10.1136/bmjopen-2014-004952published Online First: Epub Date]|.
33. *British National Formulary London*: British Medical Association and Royal Pharmaceutical Society of Great Britain, 2013.
34. Gupta GP, Massague J. Cancer metastasis: building a framework. Cell 2006;**127**(4):679-95 doi: 10.1016/j.cell.2006.11.001published Online First: Epub Date]|.
35. IARC. IARC Monographs on the evaluation of carcinogenic risks to humans. Volume 44 Alcohol drinking, 1988.
36. IARC. IARC Monographs on the evaluation of carcinogenic risks to humans. Volume 83 Tobacco smoke and involuntary smoking, 2004.
37. Reeves GK, Pirie K, Beral V, Green J, Spencer E, Bull D. Cancer incidence and mortality in relation to body mass index in the Million Women Study: cohort study. BMJ (Clinical research ed.) 2007;**335**(7630):1134 doi: 10.1136/bmj.39367.495995.AEpublished Online First: Epub Date]|.
38. National Cancer Intelligence Network and Cancer Research UK. Cancer Incidence and Survival by Major Ethnic Group, England 2002-2006., 2009.
39. Coleman RE, Rubens RD. The clinical course of bone metastases from breast cancer. Br J Cancer 1987;**55**(1):61-6
40. Yong M, Jensen AO, Jacobsen JB, Norgaard M, Fryzek JP, Sorensen HT. Survival in breast cancer patients with bone metastases and skeletal-related events: a population-based cohort study in Denmark (1999-2007). Breast Cancer Res Treat 2011;**129**(2):495-503 doi: 10.1007/s10549-011-1475-5published Online First: Epub Date]|.
41. Jensen AO, Jacobsen JB, Norgaard M, Yong M, Fryzek JP, Sorensen HT. Incidence of bone metastases and skeletal-related events in breast cancer patients: a population-based cohort study in Denmark. BMC Cancer

For peer review only

2011;**11**:29 doi: 10.1186/1471-2407-11-29published Online First: Epub Date]|.
42. Joint Epilepsy Council of the UK and Ireland. Epilepsy prevalence, incidence and other statistics. http://www.epilepsyscotland.org.uk/pdf/Joint_Epilepsy_Council_Prevalence_and_Incidence_September_11_(3).pdf 2011
43. Nicholas JM, Ridsdale L, Richardson MP, Ashworth M, Gulliford MC. Trends in antiepileptic drug utilisation in UK primary care 1993-2008: cohort study using the General Practice Research Database. Seizure : the journal of the British Epilepsy Association 2012;**21**(6):466-70 doi: 10.1016/j.seizure.2012.04.014published Online First: Epub Date]|.
44. Fang MC, Stafford RS, Ruskin JN, Singer DE. National trends in antiarrhythmic and antithrombotic medication use in atrial fibrillation. Arch Intern Med 2004;**164**(1):55-60 doi: 10.1001/archinte.164.1.55published Online First: Epub Date]|.
45. Cancer Research UK. Lifetime risk of cancer. 2014. http://www.cancerresearchuk.org/cancer-info/cancerstats/incidence/risk/statistics-on-the-risk-of-developing-cancer - Lifetime5).
46. von Elm E, Altman DG, Egger M, Pocock SJ, Gotzsche PC, Vandenbroucke JP. Strengthening the Reporting of Observational Studies in Epidemiology (STROBE) statement: guidelines for reporting observational studies. BMJ (Clinical research ed.) 2007;**335**(7624):806-8 doi: 10.1136/bmj.39335.541782.ADpublished Online First: Epub Date]|.

For peer review only

Group Name	Read code	Description
Breast cancer	B34	Malignant neoplasm of female breast
Breast cancer	B34-1	Ca female breast
Breast cancer	B34-98	Ca breast - NOS
Breast cancer	B34-99	Carcinoma breast
Breast cancer	B340	Malignant neoplasm of nipple and areola of female breast
Breast cancer	B340-99	Ca breast - nipple/central
Breast cancer	B3400	Malignant neoplasm of nipple of female breast
Breast cancer	B3401	Malignant neoplasm of areola of female breast
Breast cancer	B340z	Malignant neoplasm of nipple or areola of female breast NOS
Breast cancer	B341	Malignant neoplasm of central part of female breast
Breast cancer	B342	Malignant neoplasm of upper-inner quadrant of female breast
Breast cancer	B342-99	Ca breast-upper,inner quadrant
Breast cancer	B343	Malignant neoplasm of lower-inner quadrant of female breast
Breast cancer	B343-99	Ca breast-lower,inner quadrant
Breast cancer	B344	Malignant neoplasm of upper-outer quadrant of female breast
Breast cancer	B344-99	Ca breast-upper,outer quadrant
Breast cancer	B345	Malignant neoplasm of lower-outer quadrant of female breast
Breast cancer	B345-99	Ca breast-lower,outer quadrant
Breast cancer	B346	Malignant neoplasm of axillary tail of female breast
Breast cancer	B346-99	Ca breast - axillary tail
Breast cancer	B347	Malignant neoplasm, overlapping lesion of breast
Breast cancer	B34y	Malignant neoplasm of other site of female breast
Breast cancer	B34y0	Malignant neoplasm of ectopic site of female breast
Breast cancer	B34yz	Malignant neoplasm of other site of female breast NOS
Breast cancer	B34z	Malignant neoplasm of female breast NOS
Breast cancer	B34z-99	Ca breast - NOS
Breast cancer	B35	Malignant neoplasm of male breast
Breast cancer	B35-99	Ca breast - male
Breast cancer	B350	Malignant neoplasm of nipple and areola of male breast
Breast cancer	B3500	Malignant neoplasm of nipple of male breast
Breast cancer	B3501	Malignant neoplasm of areola of male breast
Breast cancer	B350z	Malignant neoplasm of nipple or areola of male breast NOS
Breast cancer	B35z	Malignant neoplasm of other site of male breast

For peer review only

Breast cancer	B35z0	Malignant neoplasm of ectopic site of male breast
Breast cancer	B35zz	Malignant neoplasm of male breast NOS
Breast cancer	B36	Local recurrence of malignant tumour of breast
Breast cancer	B58y0	Secondary malignant neoplasm of breast
Breast cancer	B830	Carcinoma in situ of breast
Breast cancer	B830-99	Carcinoma in situ breast
Breast cancer	B8300	Lobular carcinoma in situ of breast
Breast cancer	B8301	Intraductal carcinoma in situ of breast
Breast cancer	B933	Neoplasm of uncertain behaviour of breast
Breast cancer	BA03	Neoplasm of unspecified nature of breast
Breast cancer	BB94-1	[M]Secretory breast carcinoma
Breast cancer	Byu6	[X]Malignant neoplasm of breast
Breast cancer	EMISNQLO13	Local recurrence of malignant tumour of breast
Breast cancer	HNG0190	[RFC] Breast cancer
Breast cancer	ZV103	[V]Personal history of malignant neoplasm of breast

Group Name	Read code	Description
Colorectal cancer	B13	Malignant neoplasm of colon
Colorectal cancer	B130	Malignant neoplasm of hepatic flexure of colon
Colorectal cancer	B130-99	Ca hepatic flexure - colon
Colorectal cancer	B131	Malignant neoplasm of transverse colon
Colorectal cancer	B131-99	Ca transverse colon
Colorectal cancer	B132	Malignant neoplasm of descending colon
Colorectal cancer	B132-99	Ca descending colon
Colorectal cancer	B133	Malignant neoplasm of sigmoid colon
Colorectal cancer	B133-99	Ca sigmoid colon
Colorectal cancer	B134	Malignant neoplasm of caecum
Colorectal cancer	B134-1	Carcinoma of caecum
Colorectal cancer	B135	Malignant neoplasm of appendix
Colorectal cancer	B136	Malignant neoplasm of ascending colon
Colorectal cancer	B136-99	Ca ascending colon
Colorectal cancer	B137	Malignant neoplasm of splenic flexure of colon
Colorectal cancer	B137-99	Ca splenic flexure - colon
Colorectal cancer	B138	Malignant neoplasm, overlapping lesion of colon

For peer review only

Colorectal cancer	B139	Hereditary nonpolyposis colon cancer
Colorectal cancer	B13y	Malignant neoplasm of other specified sites of colon
Colorectal cancer	B13z	Malignant neoplasm of colon NOS
Colorectal cancer	B13z-1	Colonic cancer
Colorectal cancer	B14	Malignant neoplasm of rectum, rectosigmoid junction and anus
Colorectal cancer	B14-99	Ca rectum + Ca anus
Colorectal cancer	B140	Malignant neoplasm of rectosigmoid junction
Colorectal cancer	B140-99	Ca rectosigmoid junction
Colorectal cancer	B141	Malignant neoplasm of rectum
Colorectal cancer	B141-1	Carcinoma of rectum
Colorectal cancer	B141-2	Rectal carcinoma
Colorectal cancer	B141-99	Adenocarcinoma of rectum
Colorectal cancer	B1420	Malignant neoplasm of cloacogenic zone
Colorectal cancer	B143	Malignant neoplasm of anus unspecified
Colorectal cancer	B14y	Malig neop other site rectum, rectosigmoid junction and anus
Colorectal cancer	B14z	Malignant neoplasm rectum,rectosigmoid junction and anus NOS
Colorectal cancer	B14z-99	Ca rectum + Ca anus NOS
Colorectal cancer	B1z0-1	Cancer of bowel
Colorectal cancer	B575	Secondary malignant neoplasm of large intestine and rectum
Colorectal cancer	B5750	Secondary malignant neoplasm of colon
Colorectal cancer	B5751	Secondary malignant neoplasm of rectum
Colorectal cancer	B575z	Secondary malig neop of large intestine or rectum NOS
Colorectal cancer	B803	Carcinoma in situ of colon
Colorectal cancer	B8030	Carcinoma in situ of hepatic flexure of colon
Colorectal cancer	B8031	Carcinoma in situ of transverse colon
Colorectal cancer	B8032	Carcinoma in situ of descending colon
Colorectal cancer	B8033	Carcinoma in situ of sigmoid colon
Colorectal cancer	B8036	Carcinoma in situ of ascending colon
Colorectal cancer	B8037	Carcinoma in situ of splenic flexure of colon
Colorectal cancer	B8038	High grade dysplasia of colon
Colorectal cancer	B803z	Carcinoma in situ of colon NOS
Colorectal cancer	B804	Carcinoma in situ of rectum and rectosigmoid junction
Colorectal cancer	B8040	Carcinoma in situ of rectosigmoid junction
Colorectal cancer	B8041	Carcinoma in situ of rectum

For peer review only

Colorectal cancer	B804z	Carcinoma in situ of rectum or rectosigmoid junction NOS
Colorectal cancer	B902	Neop of uncertain behaviour stomach, intestines and rectum
Colorectal cancer	B9024	Neoplasm of uncertain behaviour of colon
Colorectal cancer	B9025	Neoplasm of uncertain behaviour of rectum
Colorectal cancer	B902z	Neop of uncertain behaviour stomach, intestine or rectum NOS
Colorectal cancer	BB5N	[M]Adenomatous and adenocarcinomatous polyps of colon
Colorectal cancer	EGTON2B152	Cause of Death- Ca Rectum
Colorectal cancer	EMISNQHE41	Hereditary nonpolyposis colon cancer
Colorectal cancer	HNG0201	[RFC] Cancer of the rectum

Group Name	Read code	Description
Prostate cancer	14270	H/O: prostate cancer
Prostate cancer	1J08	Suspected prostate cancer
Prostate cancer	4M0	Gleason grading of prostate cancer
Prostate cancer	4M00	Gleason prostate grade 2-4 (low)
Prostate cancer	4M01	Gleason prostate grade 5-7 (medium)
Prostate cancer	4M02	Gleason prostate grade 8-10 (high)
Prostate cancer	B46	Malignant neoplasm of prostate
Prostate cancer	B58y5	Secondary malignant neoplasm of prostate
Prostate cancer	B7C20	Adenoma of prostate
Prostate cancer	B834	Carcinoma in situ of prostate
Prostate cancer	B8340	High grade prostatic intraepithelial neoplasia
Prostate cancer	B8341	Prostatic intraepithelial neoplasia
Prostate cancer	B915	Neoplasm of uncertain behaviour of prostate
Prostate cancer	EMISNQPR140	Prostatic intraepithelial neoplasia
Prostate cancer	HNG0200	[RFC] Cancer of the prostate
Prostate cancer	K223	Dysplasia of prostate
Prostate cancer	PCSDT1PR16	Prostate carcinoma
Prostate cancer	ZV104-5	[V]Personal history of malignant neoplasm of prostate

Group Name	Read code	Description
Alcohol status	136	Alcohol consumption
Alcohol status	1361	Teetotaller
Alcohol status	1361-1	Non drinker alcohol

For peer review only

Alcohol status	1361-2	Non-drinker alcohol
Alcohol status	1362	Trivial drinker - <1u/day
Alcohol status	1362-1	Drinks rarely
Alcohol status	1362-2	Drinks occasionally
Alcohol status	1363	Light drinker - 1-2u/day
Alcohol status	1364	Moderate drinker - 3-6u/day
Alcohol status	1365	Heavy drinker - 7-9u/day
Alcohol status	1366	Very heavy drinker - >9u/day
Alcohol status	1367	Stopped drinking alcohol
Alcohol status	1368	Alcohol consumption unknown
Alcohol status	1369	Suspect alcohol abuse - denied
Alcohol status	136A	Ex-trivial drinker (<1u/day)
Alcohol status	136a	Increasing risk drinking
Alcohol status	136B	Ex-light drinker - (1-2u/day)
Alcohol status	136b	Feels should cut down drinking
Alcohol status	136C	Ex-moderate drinker - (3-6u/d)
Alcohol status	136c	Higher risk drinking
Alcohol status	136D	Ex-heavy drinker - (7-9u/day)
Alcohol status	136d	Lower risk drinking
Alcohol status	136E	Ex-very heavy drinker-(>9u/d)
Alcohol status	136e	Declines to state current alcohol consumption
Alcohol status	136F	Spirit drinker
Alcohol status	136G	Beer drinker
Alcohol status	136H	Drinks beer and spirits
Alcohol status	136I	Drinks wine
Alcohol status	136J	Social drinker
Alcohol status	136K	Alcohol intake above recommended sensible limits
Alcohol status	136L	Alcohol intake within recommended sensible limits
Alcohol status	136M	Current non drinker
Alcohol status	136N	Light drinker
Alcohol status	136O	Moderate drinker
Alcohol status	136P	Heavy drinker
Alcohol status	136Q	Very heavy drinker
Alcohol status	136R	Binge drinker

For peer review only

Alcohol status	136S	Hazardous alcohol use
Alcohol status	136T	Harmful alcohol use
Alcohol status	136V	Alcohol units per week
Alcohol status	136X	Alcohol units consumed on heaviest drinking day
Alcohol status	136Y	Drinks in morning to get rid of hangover
Alcohol status	136Z	Alcohol consumption NOS
Alcohol status	E23-2	Alcohol problem drinking
Alcohol status	EGTON418	Alcohol intake
Alcohol status	EGTONGR6	Grade A none drinker or rare
Alcohol status	EGTONGR7	Grade B 0-20 units/week
Alcohol status	EGTONGR8	Grade C 21-49 units/week
Alcohol status	EGTONGR9	Grade D 50 or more units/week
Alcohol status	EMISNQUN23	Unsuccessful attempts to reduce alcohol consumption
Alcohol status	EMISQAL1	Alcohol AUDIT score
Alcohol status	EMISQAL2	Alcohol reduction-maintain abstinence
Alcohol status	EMISQAU1	AUDIT score <4 no drinking problem
Alcohol status	EMISQAU2	AUDIT score >5 alcohol dependence syndrome
Alcohol status	EMISQAU3	AUDIT score >5 alcohol problem drinker
Alcohol status	EMISQNO6	Not interested in reducing alcohol
Alcohol status	EMISQOT3	Other alcohol related information
Alcohol status	EMISQRE13	Ready to start reducing alcohol
Alcohol status	EMISQTH4	Thinking about reducing alcohol

**Group Name**	**Read code**	**Description**
BMI	22K	Body Mass Index
BMI	22K1	Body Mass Index normal K/M2
BMI	22K2	Body Mass Index high K/M2
BMI	22K3	Body Mass Index low K/M2
BMI	22K4	Body mass index index 25-29 - overweight
BMI	22K5	Body mass index 30+ - obesity
BMI	22K6	Body mass index less than 20
BMI	22K7	Body mass index 40+ - severely obese
BMI	22K8	Body mass index 20-24 - normal
BMI	22K9	Body mass index centile

For peer review only

BMI	22K90	Baseline body mass index centile
BMI	22KA	Target body mass index
BMI	22KB	Baseline body mass index
BMI	EMISNQBM1	BMI centile
BMI	EMISNQBO29	Body mass index less than 18.5
BMI	EMISNQTA9	Target body mass index
BMI	JHCBO5	Body mass index 18.5-24.9
BMI	PCNQBO1	Body mass index

Group Name	Read code	Description
Ethnicity	226	O/E - ethnic group
Ethnicity	226-1	O/E - ethnic origin
Ethnicity	226Z	O/E - ethnic group NOS
Ethnicity	916E	Patient ethnicity unknown
Ethnicity	918t	Carer from Black and minority ethnic group
Ethnicity	9i	Ethnic category - 2001 census
Ethnicity	9i0	British or mixed British - ethnic category 2001 census
Ethnicity	9i00	White British - ethnic category 2001 census
Ethnicity	9i1	Irish - ethnic category 2001 census
Ethnicity	9i10	White Irish - ethnic category 2001 census
Ethnicity	9i2	Other White background - ethnic category 2001 census
Ethnicity	9i20	English - ethnic category 2001 census
Ethnicity	9i21	Scottish - ethnic category 2001 census
Ethnicity	9i22	Welsh - ethnic category 2001 census
Ethnicity	9i23	Cornish - ethnic category 2001 census
Ethnicity	9i24	Northern Irish - ethnic category 2001 census
Ethnicity	9i25	Ulster Scots - ethnic category 2001 census
Ethnicity	9i26	Cypriot (part not stated) - ethnic category 2001 census
Ethnicity	9i27	Greek - ethnic category 2001 census
Ethnicity	9i28	Greek Cypriot - ethnic category 2001 census
Ethnicity	9i29	Turkish - ethnic category 2001 census
Ethnicity	9i2A	Turkish Cypriot - ethnic category 2001 census
Ethnicity	9i2B	Italian - ethnic category 2001 census
Ethnicity	9i2C	Irish Traveller - ethnic category 2001 census

For peer review only

Ethnicity	9i2D	Traveller - ethnic category 2001 census
Ethnicity	9i2E	Gypsy/Romany - ethnic category 2001 census
Ethnicity	9i2F	Polish - ethnic category 2001 census
Ethnicity	9i2J	Kosovan - ethnic category 2001 census
Ethnicity	9i2K	Albanian - ethnic category 2001 census
Ethnicity	9i2L	Bosnian - ethnic category 2001 census
Ethnicity	9i2M	Croatian - ethnic category 2001 census
Ethnicity	9i2N	Serbian - ethnic category 2001 census
Ethnicity	9i2P	Other republics former Yugoslavia - ethnic categ 2001 census
Ethnicity	9i2Q	Mixed Irish and other White - ethnic category 2001 census
Ethnicity	9i2S	Other mixed White - ethnic category 2001 census
Ethnicity	9i2T	Other White or White unspecified ethnic category 2001 census
Ethnicity	9i3	White and Black Caribbean - ethnic category 2001 census
Ethnicity	9i4	White and Black African - ethnic category 2001 census
Ethnicity	9i5	White and Asian - ethnic category 2001 census
Ethnicity	9i6	Other Mixed background - ethnic category 2001 census
Ethnicity	9i60	Black and Asian - ethnic category 2001 census
Ethnicity	9i61	Black and Chinese - ethnic category 2001 census
Ethnicity	9i62	Black and White - ethnic category 2001 census
Ethnicity	9i63	Chinese and White - ethnic category 2001 census
Ethnicity	9i64	Asian and Chinese - ethnic category 2001 census
Ethnicity	9i65	Other Mixed or Mixed unspecified ethnic category 2001 census
Ethnicity	9i7	Indian or British Indian - ethnic category 2001 census
Ethnicity	9i8	Pakistani or British Pakistani - ethnic category 2001 census
Ethnicity	9iA	Other Asian background - ethnic category 2001 census
Ethnicity	9iA1	Punjabi - ethnic category 2001 census
Ethnicity	9iA2	Kashmiri - ethnic category 2001 census
Ethnicity	9iA3	East African Asian - ethnic category 2001 census
Ethnicity	9iA4	Sri Lankan - ethnic category 2001 census
Ethnicity	9iA5	Tamil - ethnic category 2001 census
Ethnicity	9iA6	Sinhalese - ethnic category 2001 census
Ethnicity	9iA7	Caribbean Asian - ethnic category 2001 census
Ethnicity	9iA8	British Asian - ethnic category 2001 census
Ethnicity	9iA9	Mixed Asian - ethnic category 2001 census

For peer review only

Ethnicity	9iAA	Other Asian or Asian unspecified ethnic category 2001 census
Ethnicity	9iB	Caribbean - ethnic category 2001 census
Ethnicity	9iC	African - ethnic category 2001 census
Ethnicity	9iD	Other Black background - ethnic category 2001 census
Ethnicity	9iD0	Somali - ethnic category 2001 census
Ethnicity	9iD1	Nigerian - ethnic category 2001 census
Ethnicity	9iD2	Black British - ethnic category 2001 census
Ethnicity	9iD3	Mixed Black - ethnic category 2001 census
Ethnicity	9iD4	Other Black or Black unspecified ethnic category 2001 census
Ethnicity	9iE	Chinese - ethnic category 2001 census
Ethnicity	9iF	Other - ethnic category 2001 census
Ethnicity	9iF0	Vietnamese - ethnic category 2001 census
Ethnicity	9iF1	Japanese - ethnic category 2001 census
Ethnicity	9iF2	Filipino - ethnic category 2001 census
Ethnicity	9iF3	Malaysian - ethnic category 2001 census
Ethnicity	9iF4	Buddhist - ethnic category 2001 census
Ethnicity	9iF5	Hindu - ethnic category 2001 census
Ethnicity	9iF6	Jewish - ethnic category 2001 census
Ethnicity	9iF7	Muslim - ethnic category 2001 census
Ethnicity	9iF8	Sikh - ethnic category 2001 census
Ethnicity	9iF9	Arab - ethnic category 2001 census
Ethnicity	9iFA	North African - ethnic category 2001 census
Ethnicity	9iFC	Israeli - ethnic category 2001 census
Ethnicity	9iFD	Iranian - ethnic category 2001 census
Ethnicity	9iFE	Kurdish - ethnic category 2001 census
Ethnicity	9iFF	Moroccan - ethnic category 2001 census
Ethnicity	9iFG	Latin American - ethnic category 2001 census
Ethnicity	9iFH	South and Central American - ethnic category 2001 census
Ethnicity	9iFK	Any other group - ethnic category 2001 census
Ethnicity	9iG	Ethnic category not stated - 2001 census
Ethnicity	9S	Ethnic groups (census)
Ethnicity	9S12	Other white ethnic group
Ethnicity	9S14	Other white British ethnic group
Ethnicity	9SA	Other ethnic non-mixed (NMO)

For peer review only

Ethnicity	9SA1	Brit. ethnic minor. spec.(NMO)
Ethnicity	9SA2	Brit. ethnic minor. unsp (NMO)
Ethnicity	9SAD	Other ethnic NEC (NMO)
Ethnicity	9SD	Ethnic group not given - patient refused
Ethnicity	9SE	Ethnic group not recorded
Ethnicity	9SG	Other black ethnic group
Ethnicity	9SH	Other Asian ethnic group
Ethnicity	9SJ	Other ethnic group
Ethnicity	9SZ	Ethnic groups (census) NOS
Ethnicity	9T	Ethnicity and other related nationality data
Ethnicity	9T1	New Zealand ethnic groups
Ethnicity	9T11	New Zealand European
Ethnicity	9T11-1	Pakeha
Ethnicity	9T12	Other European in New Zealand
Ethnicity	9T13	New Zealand Maori
Ethnicity	9T14	Samoan
Ethnicity	9T15	Cook Island Maori
Ethnicity	9T16	Tongan
Ethnicity	9T17	Niuean
Ethnicity	9T18	Tokelauan
Ethnicity	9T19	Fijian
Ethnicity	9T1A	Other Pacific ethnic group
Ethnicity	9T1B	South East Asian
Ethnicity	9T1C	Chinese
Ethnicity	9T1D	Indian
Ethnicity	9T1E	Other Asian
Ethnicity	9T1Y	Other New Zealand ethnic group
Ethnicity	9T1Z	New Zealand ethnic group NOS
Ethnicity	9T2	Traveller - gypsy
Ethnicity	9T3	Yemeni
Ethnicity	9T4	Romanian
Ethnicity	9T5	Bulgarian
Ethnicity	9T6	Czech
Ethnicity	9T7	Slovak

For peer review only

Ethnicity	9T8	Portuguese
Ethnicity	9T9	Nepali
Ethnicity	EMISNQBR14	Brazilian
Ethnicity	EMISNQPO8	Portuguese

Group Name	Read code	Description
Metastases	1D18	Pain from metastases
Metastases	B153	Secondary malignant neoplasm of liver
Metastases	B56	Secondary and unspecified malignant neoplasm of lymph nodes
Metastases	B56-1	Lymph node metastases
Metastases	B560	Secondary and unspec malig neop lymph nodes head/face/neck
Metastases	B560-99	Secondary nodes - head/neck
Metastases	B5600	Secondary and unspec malig neop of superficial parotid LN
Metastases	B5601	Secondary and unspec malignant neoplasm mastoid lymph nodes
Metastases	B5602	Secondary and unspec malig neop superficial cervical LN
Metastases	B5603	Secondary and unspec malignant neoplasm occipital lymph node
Metastases	B5604	Secondary and unspec malig neop deep parotid lymph nodes
Metastases	B5605	Secondary and unspec malig neop submandibular lymph nodes
Metastases	B5606	Secondary and unspec malig neop of facial lymph nodes
Metastases	B5607	Secondary and unspec malig neop submental lymph nodes
Metastases	B5608	Secondary and unspec malig neop anterior cervical LN
Metastases	B5609	Secondary and unspec malig neop deep cervical LN
Metastases	B560z	Secondary unspec malig neop lymph nodes head/face/neck NOS
Metastases	B561	Secondary and unspec malig neop intrathoracic lymph nodes
Metastases	B561-99	Secondary nodes -intrathoracic
Metastases	B5610	Secondary and unspec malig neop internal mammary lymph nodes
Metastases	B5611	Secondary and unspec malig neop intercostal lymph nodes
Metastases	B5612	Secondary and unspec malig neop diaphragmatic lymph nodes
Metastases	B5613	Secondary and unspec malig neop ant mediastinal lymph nodes
Metastases	B5614	Secondary and unspec malig neop post mediastinal lymph nodes
Metastases	B5615	Secondary and unspec malig neop paratracheal lymph nodes
Metastases	B5616	Secondary and unspec malig neop superfic tracheobronchial LN
Metastases	B5617	Secondary and unspec malig neop inferior tracheobronchial LN
Metastases	B5618	Secondary and unspec malig neop bronchopulmonary lymph nodes

For peer review only

Metastases	B5619	Secondary and unspec malig neop pulmonary lymph nodes
Metastases	B561z	Secondary and unspec malig neop intrathoracic LN NOS
Metastases	B562	Secondary and unspec malig neop intra-abdominal lymph nodes
Metastases	B562-99	Secondary nodes - intra.abdom.
Metastases	B5620	Secondary and unspec malig neop coeliac lymph nodes
Metastases	B5621	Secondary and unspec malig neop superficial mesenteric LN
Metastases	B5622	Secondary and unspec malig neop inferior mesenteric LN
Metastases	B5623	Secondary and unspec malig neop common iliac lymph nodes
Metastases	B5624	Secondary and unspec malig neop external iliac lymph nodes
Metastases	B562z	Secondary and unspec malig neop intra-abdominal LN NOS
Metastases	B563	Secondary and unspec malig neop axilla and upper limb LN
Metastases	B563-99	Secondary nodes - axilla/arm
Metastases	B5630	Secondary and unspec malig neop axillary lymph nodes
Metastases	B5631	Secondary and unspec malig neop supratrochlear lymph nodes
Metastases	B5632	Secondary and unspec malig neop infraclavicular lymph nodes
Metastases	B5633	Secondary and unspec malig neop pectoral lymph nodes
Metastases	B563z	Secondary and unspec malig neop axilla and upper limb LN NOS
Metastases	B564	Secondary and unspec malig neop inguinal and lower limb LN
Metastases	B564-99	Secondary nodes - inguinal/leg
Metastases	B5640	Secondary and unspec malig neop superficial inguinal LN
Metastases	B5641	Secondary and unspec malig neop deep inguinal lymph nodes
Metastases	B5642	Secondary and unspec malig neop popliteal lymph nodes
Metastases	B564z	Secondary and unspec malig neop of inguinal and leg LN NOS
Metastases	B565	Secondary and unspec malig neop intrapelvic lymph nodes
Metastases	B565-99	Secondary nodes - intrapelvic
Metastases	B5650	Secondary and unspec malig neop internal iliac lymph nodes
Metastases	B5651	Secondary and unspec malig neop inferior epigastric LN
Metastases	B5652	Secondary and unspec malig neop circumflex iliac LN
Metastases	B5653	Secondary and unspec malig neop sacral lymph nodes
Metastases	B5654	Secondary and unspec malig neop obturator lymph nodes
Metastases	B565z	Secondary and unspec malig neop intrapelvic LN NOS
Metastases	B56y	Secondary and unspec malig neop lymph nodes multiple sites
Metastases	B56y-99	Secondary nodes - multiple
Metastases	B56z	Secondary and unspec malig neop lymph nodes NOS

For peer review only

Metastases	B56z-99	Secondary nodes NOS
Metastases	B57-1	Metastases of respiratory and/or digestive systems
Metastases	B570	Secondary malignant neoplasm of lung
Metastases	B571	Secondary malignant neoplasm of mediastinum
Metastases	B572	Secondary malignant neoplasm of pleura
Metastases	B573	Secondary malignant neoplasm of other respiratory organs
Metastases	B574	Secondary malignant neoplasm of small intestine and duodenum
Metastases	B5740	Secondary malignant neoplasm of duodenum
Metastases	B5741	Secondary malignant neoplasm of jejunum
Metastases	B5742	Secondary malignant neoplasm of ileum
Metastases	B574z	Secondary malig neop of small intestine or duodenum NOS
Metastases	B575	Secondary malignant neoplasm of large intestine and rectum
Metastases	B5750	Secondary malignant neoplasm of colon
Metastases	B5751	Secondary malignant neoplasm of rectum
Metastases	B575z	Secondary malig neop of large intestine or rectum NOS
Metastases	B5760	Secondary malignant neoplasm of retroperitoneum
Metastases	B5761	Secondary malignant neoplasm of peritoneum
Metastases	B577	Secondary malignant neoplasm of liver
Metastases	B577-1	Liver metastases
Metastases	B57y	Secondary malignant neoplasm of other digestive organ
Metastases	B58	Secondary malignant neoplasm of other specified sites
Metastases	B580	Secondary malignant neoplasm of kidney
Metastases	B5823	Secondary malignant neoplasm of skin of trunk
Metastases	B5826	Secondary malignant neoplasm of skin of breast
Metastases	B583	Secondary malignant neoplasm of brain and spinal cord
Metastases	B583-99	Secondary Ca brain/spinal cord
Metastases	B5830	Secondary malignant neoplasm of brain
Metastases	B5831	Secondary malignant neoplasm of spinal cord
Metastases	B5832	Cerebral metastasis
Metastases	B583z	Secondary malignant neoplasm of brain or spinal cord NOS
Metastases	B584	Secondary malignant neoplasm of other part of nervous system
Metastases	B585	Secondary malignant neoplasm of bone and bone marrow
Metastases	B585-99	Secondary Ca bone/bone marrow
Metastases	B5850	Pathological fracture due to metastatic bone disease

For peer review only

Metastases	B586	Secondary malignant neoplasm of ovary
Metastases	B587	Secondary malignant neoplasm of adrenal gland
Metastases	B58y	Secondary malignant neoplasm of other specified sites
Metastases	B58y0	Secondary malignant neoplasm of breast
Metastases	B58y5	Secondary malignant neoplasm of prostate
Metastases	B58yz	Secondary malignant neoplasm of other specified site NOS
Metastases	B58z	Secondary malignant neoplasm of other specified site NOS
Metastases	B58z-99	Secondary Ca NOS
Metastases	B594	Secondary malignant neoplasm of unknown site
Metastases	ByuC	[X]Malignant neoplasm of ill-defined, secondary and unspeci
Metastases	ByuC3	[X]Secondary malignant neoplasm/oth+unspc respiratory organs
Metastases	ByuC4	[X]Secondary malignant neoplasm/oth+unspcfd digestive organs
Metastases	ByuC7	[X]Secondary malignant neoplasm of other specified sites
Metastases	EMISNQME41	Metastasis stage M0
Metastases	EMISNQME42	Metastasis stage M1
Metastases	EMISNQME43	Metastasis stage M2
Metastases	EMISNQME44	Metastasis stage M3
Metastases	EMISNQME45	Metastasis stage M4
Metastases	EMISNQME46	Metastasis stage M1a
Metastases	EMISNQME47	Metastasis stage M1b
Metastases	EMISNQME48	Metastasis stage M1c

Group Name	Read code	Description
Smoking status	137-1	Smoker - amount smoked
Smoking status	1371	Never smoked tobacco
Smoking status	1371-1	Non-smoker
Smoking status	1372	Trivial smoker - < 1 cig/day
Smoking status	1372-1	Occasional smoker
Smoking status	1373	Light smoker - 1-9 cigs/day
Smoking status	1374	Moderate smoker - 10-19 cigs/d
Smoking status	1375	Heavy smoker - 20-39 cigs/day
Smoking status	1376	Very heavy smoker - 40+cigs/d
Smoking status	1377	Ex-trivial smoker (<1/day)
Smoking status	1378	Ex-light smoker (1-9/day)

For peer review only

Smoking status	1379	Ex-moderate smoker (10-19/day)
Smoking status	137A	Ex-heavy smoker (20-39/day)
Smoking status	137a	Pipe tobacco consumption
Smoking status	137B	Ex-very heavy smoker (40+/day)
Smoking status	137b	Ready to stop smoking
Smoking status	137C	Keeps trying to stop smoking
Smoking status	137c	Thinking about stopping smoking
Smoking status	137D	Admitted tobacco cons untrue ?
Smoking status	137d	Not interested in stopping smoking
Smoking status	137E	Tobacco consumption unknown
Smoking status	137e	Smoking restarted
Smoking status	137F	Ex-smoker - amount unknown
Smoking status	137f	Reason for restarting smoking
Smoking status	137F-99	EX-Smoker NOS
Smoking status	137G	Trying to give up smoking
Smoking status	137g	Cigarette pack-years
Smoking status	137H	Pipe smoker
Smoking status	137h	Minutes from waking to first tobacco consumption
Smoking status	137I	Passive smoker
Smoking status	137i	Ex-tobacco chewer
Smoking status	137I0	Exposed to tobacco smoke at home
Smoking status	137J	Cigar smoker
Smoking status	137j	Ex-cigarette smoker
Smoking status	137K	Stopped smoking
Smoking status	137k	Refusal to give smoking status
Smoking status	137K0	Recently stopped smoking
Smoking status	137L	Current non-smoker
Smoking status	137l	Ex roll-up cigarette smoker
Smoking status	137L-99	Tobacco Consumption Nil
Smoking status	137M	Rolls own cigarettes
Smoking status	137m	Failed attempt to stop smoking
Smoking status	137N	Ex pipe smoker
Smoking status	137n	Total time smoked
Smoking status	137O	Ex cigar smoker

For peer review only

Smoking status	137o	Waterpipe tobacco consumption
Smoking status	137P	Cigarette smoker
Smoking status	137P-1	Smoker
Smoking status	137Q	Smoking started
Smoking status	137Q-1	Smoking restarted
Smoking status	137R	Current smoker
Smoking status	137S	Ex smoker
Smoking status	137T	Date ceased smoking
Smoking status	137U	Not a passive smoker
Smoking status	137V	Smoking reduced
Smoking status	137W	Chews tobacco
Smoking status	137X	Cigarette consumption
Smoking status	137Y	Cigar consumption
Smoking status	137Z	Tobacco consumption NOS
Smoking status	13p	Smoking cessation milestones
Smoking status	13p0	Negotiated date for cessation of smoking
Smoking status	13p1	Smoking status at 4 weeks
Smoking status	13p2	Smoking status between 4 and 52 weeks
Smoking status	13p3	Smoking status at 52 weeks
Smoking status	13p4	Smoking free weeks
Smoking status	13p7	Smoking status at 12 weeks
Smoking status	13WF-1	Smoker in the family
Smoking status	9ko	Current smoker annual review - enhanced services admin
Smoking status	9ko-1	Current smoker annual review
Smoking status	ASDFGNO1	Non Smoker - Nos
Smoking status	EGTON1024	Rolls own cigarettes
Smoking status	EGTON1025	Current Smoker NOS
Smoking status	EGTON1026	Ex-cigar smoker
Smoking status	EGTON1027	Ex- Rolled Tobacco Smoker
Smoking status	EGTON1028	Ex-smoker NOS
Smoking status	EGTON320	Smoking Status
Smoking status	EGTON321	Cigarette smoker
Smoking status	EGTON322	Ex-Cigarette Smoker
Smoking status	EGTON324	Ex-pipe smoker

For peer review only

Smoking status	EGTON326	Current smoker
Smoking status	EGTON327	Past smoker
Smoking status	EGTON328	Date stopped smoking
Smoking status	EGTONGR11	Grade B light smoker (1-10/day)
Smoking status	EGTONGR12	Grade C moderate smoker (11-20/day)
Smoking status	EGTONGR13	Grade D heavy smoker (>20 Day)
Smoking status	EGTONSM2	Smoking Age
Smoking status	EGTONSM3	Smoking Age Started
Smoking status	EGTONSM4	Smoking Age Ceased
Smoking status	EGTONSM6	Smoking clinic
Smoking status	EMISNQEX4	Ex-tobacco chewer
Smoking status	EMISNQPR396	Primary carer current smoker
Smoking status	EMISNQSM14	Smoking increased
Smoking status	EMISNQSN1	Snuff use
Smoking status	EMISNQTO12	Total time smoked
Smoking status	EMISNQWA11	Waterpipe tobacco consumption
Smoking status	EMISOTS1	Other smoking information
Smoking status	EMISQCO3	Carbon monoxide validation confirms smoker
Smoking status	EMISQDA1	Date of last cigarette
Smoking status	EMISQGR1	Gradual smoking reduction
Smoking status	EMISSMRE1	Smoker (Read codes)
Smoking status	PCSDT1DE9	Dependent smoker
Smoking status	PCSDT1HE1	Heavy smoker review due

For peer review only

STROBE Statement—checklist of items that should be included in reports of observational studies

	Item No	Recommendation
**Title and abstract**	1	(*a*) Indicate the study's design with a commonly used term in the title or the abstract
		(*b*) Provide in the abstract an informative and balanced summary of what was done and what was found
**Introduction**		
Background/rationale	2	Explain the scientific background and rationale for the investigation being reported
Objectives	3	State specific objectives, including any prespecified hypotheses
**Methods**		
Study design	4	Present key elements of study design early in the paper
Setting	5	Describe the setting, locations, and relevant dates, including periods of recruitment, exposure, follow-up, and data collection
Participants	6	(*a*) *Cohort study*—Give the eligibility criteria, and the sources and methods of selection of participants. Describe methods of follow-up<br>*Case-control study*—Give the eligibility criteria, and the sources and methods of case ascertainment and control selection. Give the rationale for the choice of cases and controls<br>*Cross-sectional study*—Give the eligibility criteria, and the sources and methods of selection of participants
		(*b*) *Cohort study*—For matched studies, give matching criteria and number of exposed and unexposed<br>*Case-control study*—For matched studies, give matching criteria and the number of controls per case
Variables	7	Clearly define all outcomes, exposures, predictors, potential confounders, and effect modifiers. Give diagnostic criteria, if applicable
Data sources/ measurement	8*	For each variable of interest, give sources of data and details of methods of assessment (measurement). Describe comparability of assessment methods if there is more than one group
Bias	9	Describe any efforts to address potential sources of bias
Study size	10	Explain how the study size was arrived at
Quantitative variables	11	Explain how quantitative variables were handled in the analyses. If applicable, describe which groupings were chosen and why
Statistical methods	12	(*a*) Describe all statistical methods, including those used to control for confounding
		(*b*) Describe any methods used to examine subgroups and interactions
		(*c*) Explain how missing data were addressed
		(*d*) *Cohort study*—If applicable, explain how loss to follow-up was addressed<br>*Case-control study*—If applicable, explain how matching of cases and controls was addressed<br>*Cross-sectional study*—If applicable, describe analytical methods taking account of sampling strategy
		(*e*) Describe any sensitivity analyses

Continued on next page

**Results**			
Participants	13*	(a) Report numbers of individuals at each stage of study—eg numbers potentially eligible, examined for eligibility, confirmed eligible, included in the study, completing follow-up, and analysed	✓
		(b) Give reasons for non-participation at each stage	✓
		(c) Consider use of a flow diagram	✓
Descriptive data	14*	(a) Give characteristics of study participants (eg demographic, clinical, social) and information on exposures and potential confounders	✓
		(b) Indicate number of participants with missing data for each variable of interest	N/A
		(c) *Cohort study*—Summarise follow-up time (eg, average and total amount)	✓
Outcome data	15*	*Cohort study*—Report numbers of outcome events or summary measures over time	✓
		Case-control study—Report numbers in each exposure category, or summary measures of exposure	
		Cross-sectional study—Report numbers of outcome events or summary measures	
Main results	16	(*a*) Give unadjusted estimates and, if applicable, confounder-adjusted estimates and their precision (eg, 95% confidence interval). Make clear which confounders were adjusted for and why they were included	✓
		(*b*) Report category boundaries when continuous variables were categorized	✓
		(*c*) If relevant, consider translating estimates of relative risk into absolute risk for a meaningful time period	
Other analyses	17	Report other analyses done—eg analyses of subgroups and interactions, and sensitivity analyses	✓
**Discussion**			
Key results	18	Summarise key results with reference to study objectives	✓
Limitations	19	Discuss limitations of the study, taking into account sources of potential bias or imprecision. Discuss both direction and magnitude of any potential bias	✓
Interpretation	20	Give a cautious overall interpretation of results considering objectives, limitations, multiplicity of analyses, results from similar studies, and other relevant evidence	N/A
Generalisability	21	Discuss the generalisability (external validity) of the study results	✓
**Other information**			
Funding	22	Give the source of funding and the role of the funders for the present study and, if applicable, for the original study on which the present article is based	✓

*Give information separately for cases and controls in case-control studies and, if applicable, for exposed and unexposed groups in cohort and cross-sectional studies.

Note: An Explanation and Elaboration article discusses each checklist item and gives methodological background and published examples of transparent reporting. The STROBE checklist is best used in conjunction with this article (freely available on the Web sites of PLoS Medicine at http://www.plosmedicine.org/, Annals of Internal Medicine at http://www.annals.org/, and Epidemiology at http://www.epidem.com/). Information on the STROBE Initiative is available at www.strobe-statement.org.

BMJ Open

Exposure to sodium channel-inhibiting drugs and cancer survival: protocol for a cohort study using the QResearch primary care database

Journal:	*BMJ Open*
Manuscript ID:	bmjopen-2014-006604.R1
Article Type:	Protocol
Date Submitted by the Author:	24-Sep-2014
Complete List of Authors:	Fairhurst, Caroline; University of York, Health Sciences<br>Watt, Ian; University of York, Health Sciences<br>Martin, Fabiola; Hull York Medical School,<br>Bland, Martin; University of York, Health Sciences<br>Brackenbury, William; University of York, Biology
**Primary Subject Heading**:	Oncology
Secondary Subject Heading:	Epidemiology, Pharmacology and therapeutics
Keywords:	PRIMARY CARE, Epilepsy < NEUROLOGY, Breast tumours < ONCOLOGY, Epidemiology < ONCOLOGY, STATISTICS & RESEARCH METHODS, Clinical trials < THERAPEUTICS

SCHOLARONE™
Manuscripts

For peer review only

Exposure to sodium channel-inhibiting drugs and cancer survival: protocol for a cohort study using the QResearch primary care database

Caroline Fairhurst[1], Ian Watt[1,2], Fabiola Martin[2,3], Martin Bland[1], and William J. Brackenbury[3*]

[1]Department of Health Sciences, University of York, York, UK

[2]Hull York Medical School, York, UK

[3]Department of Biology, University of York, York, UK

*Corresponding author:

William J. Brackenbury

Department of Biology, University of York, Heslington, York, YO10 5DD, UK

E-mail: william.brackenbury@york.ac.uk

Telephone: +44 (0) 1904 328 284

Keywords: Anticonvulsants / Breast neoplasms / Colonic neoplasms / Prostatic neoplasms / Sodium channels

Word count, excluding title page, abstract, references, figures and tables: 2292.

For peer review only

ABSTRACT

Introduction: Metastasis from solid tumours is associated with significant morbidity and mortality, and is the leading cause of cancer-related deaths. Voltage-gated sodium channels (VGSCs) are drug targets for the treatment of epilepsy. VGSCs are also present in cancer cells, where they regulate metastatic cell behaviours, including cellular movement and invasion. Treating cancer cells with the VGSC-inhibiting anticonvulsant phenytoin reduces cellular invasion and migration. Together, these suggest that VGSCs may be useful targets for inhibiting metastasis. The purpose of this study is to test the hypothesis that use of VGSC-inhibiting drugs will reduce metastasis, and therefore increase survival time in cancer patients.

Methods and analysis: A cohort study based on primary care data from the QResearch database will include patients with one of three common tumours: breast, bowel, and prostate. The primary outcome will be overall survival from date of cancer diagnosis. Cox proportional hazards regression will be used to compare the survival of cancer patients taking VGSC-inhibiting drugs (including anticonvulsants and Class I antiarrhythmic agents) with cancer patients not exposed to these drugs, adjusting for age and sex. Exposure to VGSC-inhibiting drugs will be defined as having at least one prescription for these drugs prior to cancer diagnosis. High and low exposure groups will be identified based on length of use. A number of sensitivity and secondary analyses will be conducted.

Ethics and dissemination: The protocol has been independently peer-reviewed and approved by the QResearch Scientific Board. The project has also been approved by the University of York Ethical Review Process. The results will be presented at international conferences and published in an open access peer-reviewed journal, in accordance with the STROBE criteria.

For peer review only

STRENGTHS AND LIMITATIONS OF THIS STUDY

- Primary care research data
- Large sample size and statistical power
- Planned sensitivity analyses
- Prescription-based study
- No direct information on metastasis, estimation is *via* overall survival
- Some variables of interest may be missing and/or poor quality in GP data

INTRODUCTION

Bowel, breast and colon cancer are common cancers, which if diagnosed late have often already spread to secondary sites (metastasised). Metastasis is associated with significant morbidity and mortality. Metastasis is the leading cause of cancer-related deaths[1] because a metastatic cancer is rarely amenable to cure, and interventions are largely limited to palliation.[2] Therefore, there is an urgent need to identify and/or develop new metastasis prevention strategies.

The classical role of voltage-gated sodium channels (VGSCs) is to transmit action potentials in electrically excitable cells, e.g. neurons and cardiomyocytes.[3] VGSCs also regulate neuronal growth and migration.[4-7] Related to these functions, VGSCs are clinical targets for a range of disorders, including epilepsy, cardiac arrhythmias, neuropathic pain and depression.[8] The mode of action of a number of commonly prescribed antiepileptic drugs (anticonvulsants), including phenytoin, lamotrigine, carbamazepine and valproate, is to inhibit VGSCs.[9] Similarly, the principal mode of action of Class I antiarrhythmic drugs is to inhibit VGSCs.[10]

For peer review only

More recently, VGSCs have been identified in cells from a number of major cancers, including carcinomas of the breast, prostate and colon.[11 12] In these cells, VGSCs promote *in vitro* cellular behaviours that are associated with metastasis, including migration and invasion.[13-18] Overexpression of the VGSC β1 subunit in breast cancer cells increases metastasis in mice.[19-21] The VGSC-inhibiting anticonvulsant phenytoin significantly reduces migration and invasion of metastatic breast and prostate cancer cells *in vitro*.[22 23] Together, these data suggest that VGSCs may be useful targets for anti-metastatic therapy, and that VGSC-inhibiting drugs may improve survival from certain cancers.[11 24] Although the effect of several anticonvulsants on risk of developing various cancers has been studied before (reviewed in[25]), the relationship between VGSC-inhibiting drugs and survival of cancer patients has not been investigated.

The purpose of this study is to test the hypothesis that use of VGSC-inhibiting drugs will predict increased time to metastasis and thus improved survival time in cancer patients. The objectives are to investigate:

- The relationship between use of all VGSC-inhibiting (anticonvulsant and Class I antiarrhythmic) drugs and overall survival of cancer patients. We will focus on carcinomas of the breast, colon and prostate because they are the most common and VGSC expression has been extensively studied in these tumours.[11 13-17 26-30]
- The relationship between use of all VGSC-inhibiting drugs and cancer specific survival.
- The relationship between individual VGSC-inhibiting drugs and overall survival.

For peer review only

There are no systematic reviews exploring this area and we are addressing this gap by conducting a review concurrent to this study [PROSPERO registration number CRD42014013574].

METHODS AND ANALYSIS

Data source and sample selection

This study will use general practice data accessed from QResearch (http://www.qresearch.org), a large consolidated database derived from the anonymised health records of over 13 million patients from 753 general practices (representing around 7% of UK practices). QResearch data are collected from the EMIS GP computer system and have been validated using other sources and shown to yield similar results to other databases, e.g. the Clinical Practice Research Datalink (CPRD).[31 32] QResearch has been used previously to study associations between cancer and prescription information.[31]

An open cohort of 100,000 patients (aged 30 years or older) with a diagnosis of breast, colorectal or prostate cancer will be identified who were registered with a QResearch practice during the study period between 1st January 1998 and 31st December 2013. This will include all those cancer patients in the database who have a prescription of one of the index drugs recorded before their date of cancer diagnosis (Table 1).[33] The remaining patients will be randomly selected controls. Time from date of diagnosis to death will be investigated and data will be right-censored in patients who are still alive at the end of the study period. Cancer diagnoses will be based on Read code information (available online at clinicalcodes.org/medcodes/article/17/).

For peer review only - http://bmjopen.bmj.com/site/about/guidelines.xhtml

Table 1. Voltage-gated Na^+ channel-inhibiting drugs			
Drug/derivative	Alternative names	Classification	British National Formulary (BNF) section[33]
Carbamazapine, eslicarbazepine, oxcarbazepine	Arbil, Carbagen SR, Epimaz, Inovelon, Tegretol, Teril, Timonil, Trileptal, Zebinix	Anticonvulsant	4.2.3, 4.7.3, 4.8.1
Disopyramide	Dirythmin, Isomide, Rythmodan	Class Ia antiarrhythmic	2.3.2
Flecainide	Tambocor	Class Ic antiarrhythmic	2.3.2
Lacosamide	Vimpat	Anticonvulsant	4.8.1
Lamotrigine	Lamictal	Anticonvulsant	4.8.1
Lidocaine	Lignocaine, Xylocard	Class Ib antiarrhythmic	2.3.2, 15.2
Mexiletine	Mexitil	Class Ib antiarrhythmic	2.3.2
Moracizine	Ethmozine	Class Ic antiarrhythmic	-
Phenytoin, fosphenytoin	Epanutin, Pentran	Anticonvulsant, Class Ib antiarrhythmic	4.7.3, 4.8.1, 4.8.2
Procainamide	Pronestyl	Class Ia antiarrhythmic	2.3.2
Propafenone	Arythmol	Class Ic	2.3.2

For peer review only

		antiarrhythmic	
Quinidine	Kiditard	Class Ia antiarrhythmic	-
Ranolazine	Ranexa	Antianginal	2.6.3
Riluzole	Rilutek	Treatment for amyotrophic lateral sclerosis	4.9.3
Tocainide	Tonocard	Class Ib antiarrhythmic	-
Topiramate	Topamax	Anticonvulsant	4.7.4, 4.8.1
Valproic acid, sodium valproate	Convulex, Depakote, Epilim, Epival, Episenta, Orlept	Anticonvulsant	4.2.3, 4.7.4, 4.8.1

Exclusions

Temporary residents and patients registered with QResearch within 12 months of data extraction will be excluded. Cases without diagnosis of one of the three index cancers (breast, colorectal, or prostate cancer) will be excluded. Patients with anomalous, incorrect or infeasible dates will be excluded, e.g., dates of cancer diagnoses recorded before birth or after death. We shall assume that dates of birth and death are correct. Any patient with a date of diagnosis that indicates they were younger than 25 at the time of diagnosis will be excluded as it is unlikely a person of that age would get one of these three cancers.

Exposure

For peer review only

A participant will be considered as exposed if they have had at least one prescription for one of the index drugs. Assuming continuous treatment use between prescriptions, we will identify two exposure groups: a *low* exposure group (less than 6 months worth of prescriptions) and a *moderate to high* exposure group (six months or more prescriptions). The exposed groups, separately and in combination, will be compared with the control group (cases without any prescription for one of the index drugs). Patients with one prescription for a drug that would have been used as a local anaesthetic, e.g. lidocaine, will be excluded.

Outcome measures

Metastasis is estimated to be responsible for 90% of deaths from solid tumours.[34] However, metastasis itself is not reliably recorded in GP data and so the primary outcome measure will be overall survival following cancer diagnosis as a proxy for metastasis. Secondary outcome measures will be cancer-specific survival for each index type of cancer and overall survival across each drug, numbers permitting.

Confounding factors

Data on the following confounders will be requested: age, gender, alcohol consumption, smoking status, body mass index (BMI) and ethnicity. Data on alcohol, smoking and BMI are routinely collected and as such a single patient may have multiple recorded observations for these variables assessed over time. We will consider the observations measured at the closest date before the date of cancer diagnosis, based on appropriate Read codes (available online at clinicalcodes.org/medcodes/article/17/). The patients will be categorised as follows:

For peer review only - http://bmjopen.bmj.com/site/about/guidelines.xhtml

- Alcohol consumption[35] categorised as: Non/trivial drinker (<1unit/day), Light drinker (1-2units/day), Moderate-very heavy drinker(3+units/day) and Not recorded/known.
- Smoking status[36] categorised as: Ex-smoker, Smoker, Non-smoker, and Not recorded/known.
- BMI,[37] categorised as Underweight (<18.5), Normal range [18.5-25), Overweight [25, 30), Obese (30+) and Not recorded/known.
- Ethnicity[38] categorised according to the groupings used in the 2011 UK census: White; Mixed/Multiple ethnic groups; Asian/Asian British; Black/African/Caribbean/Black British; Other Ethnic group. We shall also include a 'Not recorded/known' category.

Sample size calculation

Up to 100,000 eligible cases will be used, which is the maximum sample size that will be released by QResearch. At breast cancer diagnosis, approximately 6% of patients present with metastatic lesions, with bone being the most common site.[39] Of patients presenting without bone metastasis at diagnosis, 3.6% subsequently develop metastases.[40 41] The majority (90%) of metastases will lead to death.[34] Pharmacological blockade of VGSCs inhibits invasion of breast, colorectal and prostate cancer cells *in vitro* by 25-50%.[13 15 22 23] Therefore, assuming 3.6% of cancer diagnoses lead to a metastasis and most of these to death, with standard significance level alpha = 5% and power = 90%, we would require 4248 in the exposed group to detect a fall of 25% in the metastasis (or death) rate and 928 to detect a fall of 50%. This is based on 20 comparison patients per exposed patient, but this ratio is not critical. If we include 6% with a metastasis present at initial diagnosis, these numbers fall to 1503 and 330.

For peer review only - http://bmjopen.bmj.com/site/about/guidelines.xhtml

The prevalence of epilepsy is estimated to be 1%.[42] Together, the most commonly used VGSC-inhibiting anticonvulsants, phenytoin, lamotrigine, carbamazepine and valproate, account for >82% of all antiepileptic drug use.[43] By contrast, Class I antiarrhythmic drug use has been considerably less common: <5% in patients with cardiac arrhythmia.[44] Thus, using these data as a guide, we might reasonably anticipate that around 0.8% of cancer patients would be using one of these VGSC-inhibiting drugs. To meet our largest target sample size, 4248, we would therefore be looking for a sample that contained 530,000 people with a diagnosis of one of the target cancers. To meet the lower target of 928, we would require 116,000 diagnoses. Given that we are studying deaths rather than metastases *per se*, we will be unable to distinguish between metastases present at diagnosis and detected subsequently. Therefore, if we include 6% assumed to have a metastasis present at initial diagnosis, we would require 187,875 and 41,250 diagnoses to detect falls in metastasis of 25% and 50% respectively.

According to Cancer Research UK,[45] the lifetime risk in 2010 for the four major cancer sites was almost 13% (female breast), 6% (female lung), 8% (male lung), 6% (female bowel including anus), 7% (male bowel including anus) and 13% (prostate). Hence for our chosen sites, we expect approximately 21% of women and 20% of men to experience a positive diagnosis at some time. We will not have lifetime data for many in the database, but we might anticipate that 10% of a database sample would have a history of one of these sites. Thus the Qresearch database of 13 million people is large enough to achieve our largest sample target.

Statistical analysis

For peer review only

Analysis will be conducted in Stata v13, using two-sided significance at the 5% level. For each Cox model, only the patients with complete data for each of the covariates controlled for in the model will be included in the analysis.

Descriptive summaries

The characteristics of the comparison groups will be described using summary statistics. Categorical data will be presented as frequency and percentage, and continuous variables will be summarised using descriptive statistics (mean, standard deviation, median, 1st and 3rd quartiles, minimum and maximum). The flow of patients in the QResearch database will be presented in a diagram.

Primary analysis

The primary analysis will compare the combined exposure group to the control group. For each group, the distribution of time from diagnosis of cancer to death will be described using Kaplan-Meier survival estimates. Kaplan-Meier survival curves will be presented for the two groups. The statistical equivalence of the two curves will be tested using the log-rank test. Right censoring will occur if the patient is still alive at the end of the study period (31st December 2013). Median time to death, with a 95% confidence interval (CI) will be presented. If the estimated survivor function is greater than 0.5 throughout the study it will not be possible to estimate the median survival time and other percentiles survival values (i.e. 90%, 80%, 75%, as appropriate) will be presented.

We will compare the survival of exposed cases with control cases from time of diagnosis of one of the three index cancers using a Cox Proportional Hazards regression model. The endpoint will be all cause mortality. We will adjust the Cox

For peer review only - http://bmjopen.bmj.com/site/about/guidelines.xhtml

model for type of cancer (breast, bowel or prostate), gender and age at diagnosis. Age will be included with both a linear and quadratic term (age + age^2). We will assume that all included patients are receiving the most appropriate standard treatment for their disease, so we will not adjust for cancer-treating drug intake. Hazard ratios will be presented with p-values and 95% confidence intervals.

Cox regression assumes that the proportional hazards model applies. To assess this, we shall plot –log(-log(S(t))) against log(time), where S(t) is the survivor function at time *t*. The curves for the two groups should be parallel. We will also consider a chi-squared test of the Schoenfeld residuals to assess the null hypothesis of no relationship between the hazards in each group. If the assumptions are not met, we shall try to investigate why this is.

Sensitivity analysis

We will repeat the primary analysis, but adjust the Cox model, in turn, for confounding variables: ethnicity, body mass index, smoking and alcohol consumption.

Secondary analyses

Each of the following secondary end points will be analysed like the primary outcome (unless indicated) with identical censoring strategy:

- If cancer type proves to be a significant predictor in the primary model then we will consider cancer specific survival
- Survival of Low exposure group compared with control group
- Survival of High exposure group compared with control group

For peer review only

- Survival of combined exposure group and control group with outcome of time to death from first diagnosis of any cancer, since some patients may have a diagnosis of another cancer before one of breast, bowel or prostate (a category for 'Other' will be included in the covariate for type of cancer)
- Survival of patients dependent on the main drug class that they are exposed to (numbers permitting).

For peer review only

ETHICS AND DISSEMINATION

This protocol has been independently peer-reviewed by the QResearch Scientific Board. It has also been approved by the University of York Ethical Review Process. Only the authors will have access to the data during the study, in order to guarantee confidentiality of patient information. An article detailing the results of the study will be submitted for publication in an international peer-reviewed journal, in accordance with the Strengthening the Reporting of Observational Studies in Epidemiology (STROBE) criteria.[46] The full statistical analysis will be available from the authors after publication of the results.

AUTHORS' CONTRIBUTIONS

WB had the original idea for this study. CF and WB wrote the draft of the manuscript. IW, FM and MB contributed to the development of the idea, the study design, and revised the manuscript. All authors approved the final submitted version of the manuscript.

FUNDING STATEMENT

For peer review only - http://bmjopen.bmj.com/site/about/guidelines.xhtml

This work was supported by the Medical Research Council [Fellowship G1000508] and the Wellcome Trust [ref: 097829] through the Centre for Chronic Diseases and Disorders (C2D2) at the University of York.

COMPETING INTERESTS STATEMENT

The authors declare that they have no competing interests.

REFERENCES

1. Rugo HS. The importance of distant metastases in hormone-sensitive breast cancer. Breast 2008;**17 Suppl 1**:S3-8 doi: 10.1016/S0960-9776(08)70002-Xpublished Online First: Epub Date]|.
2. Suva LJ, Griffin RJ, Makhoul I. Mechanisms of bone metastases of breast cancer. Endocr Relat Cancer 2009;**16**(3):703-13 doi: 10.1677/ERC-09-0012published Online First: Epub Date]|.
3. Hille B. *Ionic channels of excitable membranes*. 2nd ed. Sunderland (Massachusetts): Sinauer Associates Inc., 1992.
4. Brackenbury WJ, Calhoun JD, Chen C, et al. Functional reciprocity between Na+ channel Nav1.6 and β1 subunits in the coordinated regulation of excitability and neurite outgrowth. Proc Natl Acad Sci U S A 2010;**107**(5):2283-88
5. Brackenbury WJ, Yuan Y, O'Malley HA, Parent JM, Isom LL. Abnormal neuronal patterning occurs during early postnatal brain development of Scn1b-null mice and precedes hyperexcitability. Proc Natl Acad Sci U S A 2013;**110**(3):1089-94 doi: 10.1073/pnas.1208767110published Online First: Epub Date]|.
6. Catterall WA. From ionic currents to molecular mechanisms: the structure and function of voltage-gated sodium channels. Neuron 2000;**26**(1):13-25
7. Brackenbury WJ, Davis TH, Chen C, et al. Voltage-gated Na^+ channel β1 subunit-mediated neurite outgrowth requires fyn kinase and contributes to central nervous system development in vivo. J Neurosci 2008;**28**(12):3246-56
8. Clare JJ, Tate SN, Nobbs M, Romanos MA. Voltage-gated sodium channels as therapeutic targets. Drug Discov Today 2000;**5**(11):506-20
9. Mantegazza M, Curia G, Biagini G, Ragsdale DS, Avoli M. Voltage-gated sodium channels as therapeutic targets in epilepsy and other neurological disorders. Lancet Neurol 2010;**9**(4):413-24 doi: 10.1016/S1474-4422(10)70059-4published Online First: Epub Date]|.
10. Vaughan Williams EM. The relevance of cellular to clinical electrophysiology in classifying antiarrhythmic actions. J Cardiovasc Pharmacol 1992;**20 Suppl 2**:S1-7
11. Brackenbury WJ. Voltage-gated sodium channels and metastatic disease. Channels (Austin) 2012;**6**(5):352-61 doi: 10.4161/chan.21910published Online First: Epub Date]|.
12. Brackenbury WJ, Djamgoz MB, Isom LL. An emerging role for voltage-gated Na+ channels in cellular migration: regulation of central nervous system

For peer review only - http://bmjopen.bmj.com/site/about/guidelines.xhtml

development and potentiation of invasive cancers. Neuroscientist 2008;**14**(6):571-83 doi: 10.1177/1073858408320293published Online First: Epub Date]|.
13. House CD, Vaske CJ, Schwartz A, et al. Voltage-gated Na+ channel SCN5A is a key regulator of a gene transcriptional network that controls colon cancer invasion. Cancer Res 2010;**70**(17):6957-67 doi: 10.1158/0008-5472.CAN-10-1169published Online First: Epub Date]|.
14. Grimes JA, Fraser SP, Stephens GJ, et al. Differential expression of voltage-activated Na^+ currents in two prostatic tumour cell lines: contribution to invasiveness in vitro. FEBS Lett 1995;**369**(2-3):290-4
15. Fraser SP, Diss JK, Chioni AM, et al. Voltage-gated sodium channel expression and potentiation of human breast cancer metastasis. Clin Cancer Res 2005;**11**(15):5381-89 doi: 10.1158/1078-0432.CCR-05-0327published Online First: Epub Date]|.
16. Brackenbury WJ, Chioni AM, Diss JK, Djamgoz MB. The neonatal splice variant of Nav1.5 potentiates in vitro metastatic behaviour of MDA-MB-231 human breast cancer cells. Breast Cancer Res. Treat. 2007;**101**(2):149-60
17. Brackenbury WJ, Djamgoz MB. Activity-dependent regulation of voltage-gated Na^+ channel expression in Mat-LyLu rat prostate cancer cell line. J Physiol 2006;**573**(Pt 2):343-56 doi: 10.1113/jphysiol.2006.106906published Online First: Epub Date]|.
18. Brackenbury WJ, Djamgoz MB. Nerve growth factor enhances voltage-gated Na+ channel activity and Transwell migration in Mat-LyLu rat prostate cancer cell line. J Cell Physiol 2007;**210**(3):602-8 doi: 10.1002/jcp.20846published Online First: Epub Date]|.
19. Nelson M, Millican-Slater R, Forrest LC, Brackenbury WJ. The sodium channel beta1 subunit mediates outgrowth of neurite-like processes on breast cancer cells and promotes tumour growth and metastasis. Int J Cancer 2014;**135**(10):2338-51 doi: 10.1002/ijc.28890published Online First: Epub Date]|.
20. Brackenbury WJ, Isom LL. Na Channel beta Subunits: Overachievers of the Ion Channel Family. Frontiers in pharmacology 2011;**2**:53 doi: 10.3389/fphar.2011.00053published Online First: Epub Date]|.
21. Chioni AM, Brackenbury WJ, Calhoun JD, Isom LL, Djamgoz MB. A novel adhesion molecule in human breast cancer cells: voltage-gated Na+ channel beta1 subunit. Int J Biochem Cell Biol 2009;**41**(5):1216-27 doi: 10.1016/j.biocel.2008.11.001published Online First: Epub Date]|.
22. Fraser SP, Salvador V, Manning EA, et al. Contribution of functional voltage-gated Na^+ channel expression to cell behaviors involved in the metastatic cascade in rat prostate cancer: I. lateral motility. J Cell Physiol 2003;**195**(3):479-87
23. Yang M, Kozminski DJ, Wold LA, et al. Therapeutic potential for phenytoin: targeting Na(v)1.5 sodium channels to reduce migration and invasion in metastatic breast cancer. Breast Cancer Res Treat 2012;**134**(2):603-15 doi: 10.1007/s10549-012-2102-9published Online First: Epub Date]|.
24. Brackenbury WJ, Isom LL. Voltage-gated Na+ channels: potential for beta subunits as therapeutic targets. Expert Opin Ther Targets 2008;**12**(9):1191-203 doi: 10.1517/14728222.12.9.1191published Online First: Epub Date]|.
25. Singh G, Driever PH, Sander JW. Cancer risk in people with epilepsy: the role of antiepileptic drugs. Brain 2005;**128**(Pt 1):7-17 doi: 10.1093/brain/awh363published Online First: Epub Date]|.
26. Brisson L, Driffort V, Benoist L, et al. NaV1.5 Na(+) channels allosterically regulate the NHE-1 exchanger and promote the activity of breast cancer cell invadopodia. J Cell Sci 2013;**126**(Pt 21):4835-42 doi: 10.1242/jcs.123901published Online First: Epub Date]|.

For peer review only

27. Ding L, Ellis MJ, Li S, et al. Genome remodelling in a basal-like breast cancer metastasis and xenograft. Nature 2010;**464**(7291):999-1005 doi: 10.1038/nature08989published Online First: Epub Date]|.
28. Grimes JA, Djamgoz MB. Electrophysiological characterization of voltage-gated Na(+) current expressed in the highly metastatic Mat-LyLu cell line of rat prostate cancer. J Cell Physiol 1998;**175**(1):50-8
29. Gillet L, Roger S, Besson P, et al. Voltage-gated Sodium Channel Activity Promotes Cysteine Cathepsin-dependent Invasiveness and Colony Growth of Human Cancer Cells. J Biol Chem 2009;**284**(13):8680-91 doi: M806891200 [pii] 10.1074/jbc.M806891200published Online First: Epub Date]|.
30. Ding Y, Brackenbury WJ, Onganer PU, et al. Epidermal growth factor upregulates motility of Mat-LyLu rat prostate cancer cells partially via voltage-gated Na^+ channel activity. J Cell Physiol 2008;**215**(1):77-81 doi: 10.1002/jcp.21289published Online First: Epub Date]|.
31. Vinogradova Y, Coupland C, Hippisley-Cox J. Exposure to bisphosphonates and risk of gastrointestinal cancers: series of nested case-control studies with QResearch and CPRD data. BMJ (Clinical research ed.) 2013;**346**:f114 doi: 10.1136/bmj.f114published Online First: Epub Date]|.
32. Reeves D, Springate DA, Ashcroft DM, et al. Can analyses of electronic patient records be independently and externally validated? The effect of statins on the mortality of patients with ischaemic heart disease: a cohort study with nested case-control analysis. BMJ open 2014;**4**(4):e004952 doi: 10.1136/bmjopen-2014-004952published Online First: Epub Date]|.
33. *British National Formulary London*: British Medical Association and Royal Pharmaceutical Society of Great Britain, 2013.
34. Gupta GP, Massague J. Cancer metastasis: building a framework. Cell 2006;**127**(4):679-95 doi: 10.1016/j.cell.2006.11.001published Online First: Epub Date]|.
35. IARC. IARC Monographs on the evaluation of carcinogenic risks to humans. Volume 44 Alcohol drinking, 1988.
36. IARC. IARC Monographs on the evaluation of carcinogenic risks to humans. Volume 83 Tobacco smoke and involuntary smoking, 2004.
37. Reeves GK, Pirie K, Beral V, Green J, Spencer E, Bull D. Cancer incidence and mortality in relation to body mass index in the Million Women Study: cohort study. BMJ (Clinical research ed.) 2007;**335**(7630):1134 doi: 10.1136/bmj.39367.495995.AEpublished Online First: Epub Date]|.
38. National Cancer Intelligence Network and Cancer Research UK. Cancer Incidence and Survival by Major Ethnic Group, England 2002-2006., 2009.
39. Coleman RE, Rubens RD. The clinical course of bone metastases from breast cancer. Br J Cancer 1987;**55**(1):61-6
40. Yong M, Jensen AO, Jacobsen JB, Norgaard M, Fryzek JP, Sorensen HT. Survival in breast cancer patients with bone metastases and skeletal-related events: a population-based cohort study in Denmark (1999-2007). Breast Cancer Res Treat 2011;**129**(2):495-503 doi: 10.1007/s10549-011-1475-5published Online First: Epub Date]|.
41. Jensen AO, Jacobsen JB, Norgaard M, Yong M, Fryzek JP, Sorensen HT. Incidence of bone metastases and skeletal-related events in breast cancer patients: a population-based cohort study in Denmark. BMC Cancer 2011;**11**:29 doi: 10.1186/1471-2407-11-29published Online First: Epub Date]|.
42. Joint Epilepsy Council of the UK and Ireland. Epilepsy prevalence, incidence and other statistics. http://www.epilepsyscotland.org.uk/pdf/Joint_Epilepsy_Council_Prevalence_and_Incidence_September_11_(3).pdf 2011

For peer review only - http://bmjopen.bmj.com/site/about/guidelines.xhtml

43. Nicholas JM, Ridsdale L, Richardson MP, Ashworth M, Gulliford MC. Trends in antiepileptic drug utilisation in UK primary care 1993-2008: cohort study using the General Practice Research Database. Seizure : the journal of the British Epilepsy Association 2012;**21**(6):466-70 doi: 10.1016/j.seizure.2012.04.014published Online First: Epub Date]|.
44. Fang MC, Stafford RS, Ruskin JN, Singer DE. National trends in antiarrhythmic and antithrombotic medication use in atrial fibrillation. Arch Intern Med 2004;**164**(1):55-60 doi: 10.1001/archinte.164.1.55published Online First: Epub Date]|.
45. Cancer Research UK. Lifetime risk of cancer. 2014. http://www.cancerresearchuk.org/cancer-info/cancerstats/incidence/risk/statistics-on-the-risk-of-developing-cancer - Lifetime5).
46. von Elm E, Altman DG, Egger M, Pocock SJ, Gotzsche PC, Vandenbroucke JP. Strengthening the Reporting of Observational Studies in Epidemiology (STROBE) statement: guidelines for reporting observational studies. BMJ (Clinical research ed.) 2007;**335**(7624):806-8 doi: 10.1136/bmj.39335.541782.ADpublished Online First: Epub Date]|.

For peer review only

Exposure to sodium channel-inhibiting drugs and cancer survival: protocol for a cohort study using the QResearch primary care database

Caroline Fairhurst[1], Ian Watt[1,2], Fabiola Martin[2,3], Martin Bland[1], and William J. Brackenbury[3*]

[1]Department of Health Sciences, University of York, York, UK

[2]Hull York Medical School, York, UK

[3]Department of Biology, University of York, York, UK

*Corresponding author:

William J. Brackenbury

Department of Biology, University of York, Heslington, York, YO10 5DD, UK

E-mail: william.brackenbury@york.ac.uk

Telephone: +44 (0) 1904 328 284

Keywords: Anticonvulsants / Breast neoplasms / Colonic neoplasms / Prostatic neoplasms / Sodium channels

Word count, excluding title page, abstract, references, figures and tables: 2292.

For peer review only

ABSTRACT

Introduction: Metastasis from solid tumours is associated with significant morbidity and mortality, and is the leading cause of cancer-related deaths. Voltage-gated sodium channels (VGSCs) are drug targets for the treatment of epilepsy. VGSCs are also present in cancer cells, where they regulate metastatic cell behaviours, including cellular movement and invasion. Treating cancer cells with the VGSC-inhibiting anticonvulsant phenytoin reduces cellular invasion and migration. Together, these suggest that VGSCs may be useful targets for inhibiting metastasis. The purpose of this study is to test the hypothesis that use of VGSC-inhibiting drugs will reduce metastasis, and therefore increase survival time in cancer patients.

Methods and analysis: A cohort study based on primary care data from the QResearch database will include patients with one of three common tumours: breast, bowel, and prostate. The primary outcome will be overall survival from date of cancer diagnosis. Cox proportional hazards regression will be used to compare the survival of cancer patients taking VGSC-inhibiting drugs (including anticonvulsants and Class I antiarrhythmic agents) with cancer patients not exposed to these drugs, adjusting for age and sex. Exposure to VGSC-inhibiting drugs will be defined as having at least one prescription for these drugs prior to cancer diagnosis. High and low exposure groups will be identified based on length of use. A number of sensitivity and secondary analyses will be conducted.

Ethics and dissemination: The protocol has been independently peer-reviewed and approved by the QResearch Scientific Board. The project has also been approved by the University of York Ethical Review Process. The results will be presented at international conferences and published in an open access peer-reviewed journal, in accordance with the STROBE criteria.

For peer review only

STRENGTHS AND LIMITATIONS OF THIS STUDY

- Primary care research data
- Large sample size and statistical power
- Planned sensitivity analyses
- Prescription-based study
- No direct information on metastasis, estimation is *via* overall survival
- Some variables of interest may be missing and/or poor quality in GP data

INTRODUCTION

Bowel, breast and colon cancer are common cancers, which if diagnosed late have often already spread to secondary sites (metastasised). Metastasis is associated with significant morbidity and mortality. Metastasis is the leading cause of cancer-related deaths[1] because a metastatic cancer is rarely amenable to cure, and interventions are largely limited to palliation.[2] Therefore, there is an urgent need to identify and/or develop new metastasis prevention strategies.

The classical role of voltage-gated sodium channels (VGSCs) is to transmit action potentials in electrically excitable cells, e.g. neurons and cardiomyocytes.[3] VGSCs also regulate neuronal growth and migration.[4-7] Related to these functions, VGSCs are clinical targets for a range of disorders, including epilepsy, cardiac arrhythmias, neuropathic pain and depression.[8] The mode of action of a number of commonly prescribed antiepileptic drugs (anticonvulsants), including phenytoin, lamotrigine, carbamazepine and valproate, is to inhibit VGSCs.[9] Similarly, the principal mode of action of Class I antiarrhythmic drugs is to inhibit VGSCs.[10]

For peer review only

More recently, VGSCs have been identified in cells from a number of major cancers, including carcinomas of the breast, prostate and colon.[11 12] In these cells, VGSCs promote *in vitro* cellular behaviours that are associated with metastasis, including migration and invasion.[13-18] Overexpression of the VGSC β1 subunit in breast cancer cells increases metastasis in mice.[19-21] The VGSC-inhibiting anticonvulsant phenytoin significantly reduces migration and invasion of metastatic breast and prostate cancer cells *in vitro*.[22 23] ~~and has been shown to reduce metastasis in a mouse model of breast cancer (M. Nelson, M. Yang, A. A. Dowle, J. R. Thomas and W. J. Brackenbury, manuscript under review, 2014).~~ Together, these data suggest that VGSCs may be useful targets for anti-metastatic therapy, and that VGSC-inhibiting drugs may improve survival from certain cancers.[11 24] Although the effect of several anticonvulsants on risk of developing various cancers has been studied before (reviewed in[25]), the relationship between VGSC-inhibiting drugs and survival of cancer patients has not been investigated.

The purpose of this study is to test the hypothesis that use of VGSC-inhibiting drugs will predict increased time to metastasis and thus improved survival time in cancer patients. The objectives are to investigate:

- The relationship between use of all VGSC-inhibiting (anticonvulsant and Class I antiarrhythmic) drugs and overall survival of cancer patients. We will focus on carcinomas of the breast, colon and prostate because they are the most common and VGSC expression has been extensively studied in these tumours.[11 13-17 26-30]
- The relationship between use of all VGSC-inhibiting drugs and cancer specific survival.

For peer review only - http://bmjopen.bmj.com/site/about/guidelines.xhtml

- The relationship between individual VGSC-inhibiting drugs and overall survival.

There are no systematic reviews exploring this area and we are addressing this gap by conducting a review concurrent to this study [PROSPERO registration number CRD42014013574].

METHODS AND ANALYSIS

Data source and sample selection

This study will use general practice data accessed from QResearch (http://www.qresearch.org), a large consolidated database derived from the anonymised health records of over 13 million patients from 753 general practices (representing around 7% of UK practices). QResearch data are collected from the EMIS GP computer system and have been validated using other sources and shown to yield similar results to other databases, e.g. the Clinical Practice Research Datalink (CPRD).[31 32] QResearch has been used previously to study associations between cancer and prescription information.[31]

An open cohort of 100,000 patients (aged 30 years or older) with a diagnosis of breast, colorectal or prostate cancer will be identified who were registered with a QResearch practice during the study period between 1st January 1998 and 31st December 2013. This will include all those cancer patients in the database who have a prescription of one of the index drugs recorded before their date of cancer diagnosis (Table 1).[33] The remaining patients will be randomly selected controls. Time from date of diagnosis to death will be investigated and data will be right-censored in patients who are still alive at the end of the study period. Cancer

For peer review only

diagnoses will be based on Read code information (see online supplementary appendixavailable online at clinicalcodes.org/medcodes/article/17/).

Table 1. Voltage-gated Na^+ channel-inhibiting drugs

Drug/derivative	Alternative names	Classification	British National Formulary (BNF) section[33]
Carbamazapine, eslicarbazepine, oxcarbazepine	Arbil, Carbagen SR, Epimaz, Inovelon, Tegretol, Teril, Timonil, Trileptal, Zebinix	Anticonvulsant	4.2.3, 4.7.3, 4.8.1
Disopyramide	Dirythmin, Isomide, Rythmodan	Class Ia antiarrhythmic	2.3.2
Flecainide	Tambocor	Class Ic antiarrhythmic	2.3.2
Lacosamide	Vimpat	Anticonvulsant	4.8.1
Lamotrigine	Lamictal	Anticonvulsant	4.8.1
Lidocaine	Lignocaine, Xylocard	Class Ib antiarrhythmic	2.3.2, 15.2
Mexiletine	Mexitil	Class Ib antiarrhythmic	2.3.2
Moracizine	Ethmozine	Class Ic antiarrhythmic	-
Phenytoin, fosphenytoin	Epanutin, Pentran	Anticonvulsant, Class Ib antiarrhythmic	4.7.3, 4.8.1, 4.8.2

For peer review only

Procainamide	Pronestyl	Class Ia antiarrhythmic	2.3.2
Propafenone	Arythmol	Class Ic antiarrhythmic	2.3.2
Quinidine	Kiditard	Class Ia antiarrhythmic	-
Ranolazine	Ranexa	Antianginal	2.6.3
Riluzole	Rilutek	Treatment for amyotrophic lateral sclerosis	4.9.3
Tocainide	Tonocard	Class Ib antiarrhythmic	-
Topiramate	Topamax	Anticonvulsant	4.7.4, 4.8.1
Valproic acid, sodium valproate	Convulex, Depakote, Epilim, Epival, Episenta, Orlept	Anticonvulsant	4.2.3, 4.7.4, 4.8.1

Exclusions

Temporary residents and patients registered with QResearch within 12 months of data extraction will be excluded. Cases without diagnosis of one of the three index cancers (breast, colorectal, or prostate cancer) will be excluded. Patients with anomalous, incorrect or infeasible dates will be excluded, e.g., dates of cancer diagnoses recorded before birth or after death. We shall assume that dates of birth and death are correct. Any patient with a date of diagnosis that indicates they were

For peer review only

younger than 25 at the time of diagnosis will be excluded as it is unlikely a person of that age would get one of these three cancers.

Exposure

A participant will be considered as exposed if they have had at least one prescription for one of the index drugs. Assuming continuous treatment use between prescriptions, we will identify two exposure groups: a *low* exposure group (less than 6 months worth of prescriptions) and a *moderate to high* exposure group (six months or more prescriptions). The exposed groups, separately and in combination, will be compared with the control group (cases without any prescription for one of the index drugs). Patients with one prescription for a drug that would have been used as a local anaesthetic, e.g. lidocaine, will be excluded.

Outcome measures

Metastasis is estimated to be responsible for 90% of deaths from solid tumours.[34] However, metastasis itself is not reliably recorded in GP data and so the primary outcome measure will be overall survival following cancer diagnosis as a proxy for metastasis. Secondary outcome measures will be cancer-specific survival for each index type of cancer and overall survival across each drug, numbers permitting.

Confounding factors

Data on the following confounders will be requested: age, gender, alcohol consumption, smoking status, body mass index (BMI) and ethnicity. Data on alcohol, smoking and BMI are routinely collected and as such a single patient may have multiple recorded observations for these variables assessed over time. We will

For peer review only - http://bmjopen.bmj.com/site/about/guidelines.xhtml

consider the observations measured at the closest date before the date of cancer diagnosis, based on appropriate Read codes (available online at clinicalcodes.org/medcodes/article/17/detailed in online supplementary appendix).

The patients will be categorised as follows:

- Alcohol consumption[35] categorised as: Non/trivial drinker (<1unit/day), Light drinker (1-2units/day), Moderate-very heavy drinker(3+units/day) and Not recorded/known.
- Smoking status[36] categorised as: Ex-smoker, Smoker, Non-smoker, and Not recorded/known.
- BMI,[37] categorised as Underweight (<18.5), Normal range [18.5-25), Overweight [25, 30), Obese (30+) and Not recorded/known.
- Ethnicity[38] categorised according to the groupings used in the 2011 UK census: White; Mixed/Multiple ethnic groups; Asian/Asian British; Black/African/Caribbean/Black British; Other Ethnic group. We shall also include a 'Not recorded/known' category.

Sample size calculation

Up to 100,000 eligible cases will be used, which is the maximum sample size that will be released by QResearch. At breast cancer diagnosis, approximately 6% of patients present with metastatic lesions, with bone being the most common site.[39] Of patients presenting without bone metastasis at diagnosis, 3.6% subsequently develop metastases.[40 41] The majority (90%) of metastases will lead to death.[34] Pharmacological blockade of VGSCs inhibits invasion of breast, colorectal and prostate cancer cells *in vitro* by 25-50%.[13 15 22 23] Therefore, assuming 3.6% of cancer diagnoses lead to a metastasis and most of these to death, with standard significance level alpha = 5% and power = 90%, we would require 4248 in the

For peer review only - http://bmjopen.bmj.com/site/about/guidelines.xhtml

exposed group to detect a fall of 25% in the metastasis (or death) rate and 928 to detect a fall of 50%. This is based on 20 comparison patients per exposed patient, but this ratio is not critical. If we include 6% with a metastasis present at initial diagnosis, these numbers fall to 1503 and 330.

The prevalence of epilepsy is estimated to be 1%.[42] Together, the most commonly used VGSC-inhibiting anticonvulsants, phenytoin, lamotrigine, carbamazepine and valproate, account for >82% of all antiepileptic drug use.[43] By contrast, Class I antiarrhythmic drug use has been considerably less common: <5% in patients with cardiac arrhythmia.[44] Thus, using these data as a guide, we might reasonably anticipate that around 0.8% of cancer patients would be using one of these VGSC-inhibiting drugs. To meet our largest target sample size, 4248, we would therefore be looking for a sample that contained 530,000 people with a diagnosis of one of the target cancers. To meet the lower target of 928, we would require 116,000 diagnoses. Given that we are studying deaths rather than metastases *per se*, we will be unable to distinguish between metastases present at diagnosis and detected subsequently. Therefore, if we include 6% assumed to have a metastasis present at initial diagnosis, we would require 187,875 and 41,250 diagnoses to detect falls in metastasis of 25% and 50% respectively.

According to Cancer Research UK,[45] the lifetime risk in 2010 for the four major cancer sites was almost 13% (female breast), 6% (female lung), 8% (male lung), 6% (female bowel including anus), 7% (male bowel including anus) and 13% (prostate). Hence for our chosen sites, we expect approximately 21% of women and 20% of men to experience a positive diagnosis at some time. We will not have lifetime data for many in the database, but we might anticipate that 10% of a database sample would have a history of one of these sites. Thus the Qresearch database of 13 million people is large enough to achieve our largest sample target.

For peer review only - http://bmjopen.bmj.com/site/about/guidelines.xhtml

Statistical analysis

Analysis will be conducted in Stata v13, using two-sided significance at the 5% level. For each Cox model, only the patients with complete data for each of the covariates controlled for in the model will be included in the analysis.

Descriptive summaries

The characteristics of the comparison groups will be described using summary statistics. Categorical data will be presented as frequency and percentage, and continuous variables will be summarised using descriptive statistics (mean, standard deviation, median, 1st and 3rd quartiles, minimum and maximum). The flow of patients in the QResearch database will be presented in a diagram.

Primary analysis

The primary analysis will compare the combined exposure group to the control group. For each group, the distribution of time from diagnosis of cancer to death will be described using Kaplan-Meier survival estimates. Kaplan-Meier survival curves will be presented for the two groups. The statistical equivalence of the two curves will be tested using the log-rank test. Right censoring will occur if the patient is still alive at the end of the study period (31st December 2013). Median time to death, with a 95% confidence interval (CI) will be presented. If the estimated survivor function is greater than 0.5 throughout the study it will not be possible to estimate the median survival time and other percentiles survival values (i.e. 90%, 80%, 75%, as appropriate) will be presented.

For peer review only - http://bmjopen.bmj.com/site/about/guidelines.xhtml

We will compare the survival of exposed cases with control cases from time of diagnosis of one of the three index cancers using a Cox Proportional Hazards regression model. The endpoint will be all cause mortality. We will adjust the Cox model for type of cancer (breast, bowel or prostate), gender and age at diagnosis. Age will be included with both a linear and quadratic term (age + age^2). We will assume that all included patients are receiving the most appropriate standard treatment for their disease, so we will not adjust for cancer-treating drug intake. Hazard ratios will be presented with p-values and 95% confidence intervals.

Cox regression assumes that the proportional hazards model applies. To assess this, we shall plot –log(-log(S(t))) against log(time), where S(t) is the survivor function at time *t*. The curves for the two groups should be parallel. We will also consider a chi-squared test of the Schoenfeld residuals to assess the null hypothesis of no relationship between the hazards in each group. If the assumptions are not met, we shall try to investigate why this is.

Sensitivity analysis

We will repeat the primary analysis, but adjust the Cox model, in turn, for confounding variables: ethnicity, body mass index, smoking and alcohol consumption.

Secondary analyses

Each of the following secondary end points will be analysed like the primary outcome (unless indicated) with identical censoring strategy:

- If cancer type proves to be a significant predictor in the primary model then we will consider cancer specific survival

For peer review only - http://bmjopen.bmj.com/site/about/guidelines.xhtml

- Survival of Low exposure group compared with control group
- Survival of High exposure group compared with control group
- Survival of combined exposure group and control group with outcome of time to death from first diagnosis of any cancer, since some patients may have a diagnosis of another cancer before one of breast, bowel or prostate (a category for 'Other' will be included in the covariate for type of cancer)
- Survival of patients dependent on the main drug class that they are exposed to (numbers permitting).

ETHICS AND DISSEMINATION

This protocol has been independently peer-reviewed by the QResearch Scientific Board. It has also been approved by the University of York Ethical Review Process. Only the authors will have access to the data during the study, in order to guarantee confidentiality of patient information. An article detailing the results of the study will be submitted for publication in an international peer-reviewed journal, in accordance with the Strengthening the Reporting of Observational Studies in Epidemiology (STROBE) criteria.[46] The full ~~protocol and~~ statistical analysis will be available from the authors after publication of the results.

AUTHORS' CONTRIBUTIONS

WB had the original idea for this study. CF and WB wrote the draft of the manuscript. IW, FM and MB contributed to the development of the idea, the study design, and revised the manuscript. All authors approved the final submitted version of the manuscript.

For peer review only

FUNDING STATEMENT

This work was supported by the Medical Research Council [Fellowship G1000508] and the Wellcome Trust [ref: 097829] through the Centre for Chronic Diseases and Disorders (C2D2) at the University of York.

COMPETING INTERESTS STATEMENT

The authors declare that they have no competing interests.

REFERENCES

1. Rugo HS. The importance of distant metastases in hormone-sensitive breast cancer. Breast 2008;**17 Suppl 1**:S3-8 doi: 10.1016/S0960-9776(08)70002-Xpublished Online First: Epub Date]|.
2. Suva LJ, Griffin RJ, Makhoul I. Mechanisms of bone metastases of breast cancer. Endocr Relat Cancer 2009;**16**(3):703-13 doi: 10.1677/ERC-09-0012published Online First: Epub Date]|.
3. Hille B. *Ionic channels of excitable membranes*. 2nd ed. Sunderland (Massachusetts): Sinauer Associates Inc., 1992.
4. Brackenbury WJ, Calhoun JD, Chen C, et al. Functional reciprocity between Na+ channel Nav1.6 and β1 subunits in the coordinated regulation of excitability and neurite outgrowth. Proc Natl Acad Sci U S A 2010;**107**(5):2283-88
5. Brackenbury WJ, Yuan Y, O'Malley HA, Parent JM, Isom LL. Abnormal neuronal patterning occurs during early postnatal brain development of Scn1b-null mice and precedes hyperexcitability. Proc Natl Acad Sci U S A 2013;**110**(3):1089-94 doi: 10.1073/pnas.1208767110published Online First: Epub Date]|.
6. Catterall WA. From ionic currents to molecular mechanisms: the structure and function of voltage-gated sodium channels. Neuron 2000;**26**(1):13-25
7. Brackenbury WJ, Davis TH, Chen C, et al. Voltage-gated Na^+ channel β1 subunit-mediated neurite outgrowth requires fyn kinase and contributes to central nervous system development in vivo. J Neurosci 2008;**28**(12):3246-56
8. Clare JJ, Tate SN, Nobbs M, Romanos MA. Voltage-gated sodium channels as therapeutic targets. Drug Discov Today 2000;**5**(11):506-20
9. Mantegazza M, Curia G, Biagini G, Ragsdale DS, Avoli M. Voltage-gated sodium channels as therapeutic targets in epilepsy and other neurological disorders. Lancet Neurol 2010;**9**(4):413-24 doi: 10.1016/S1474-4422(10)70059-4published Online First: Epub Date]|.
10. Vaughan Williams EM. The relevance of cellular to clinical electrophysiology in classifying antiarrhythmic actions. J Cardiovasc Pharmacol 1992;**20 Suppl 2**:S1-7

For peer review only

11. Brackenbury WJ. Voltage-gated sodium channels and metastatic disease. Channels (Austin) 2012;**6**(5):352-61 doi: 10.4161/chan.21910published Online First: Epub Date]|.
12. Brackenbury WJ, Djamgoz MB, Isom LL. An emerging role for voltage-gated Na+ channels in cellular migration: regulation of central nervous system development and potentiation of invasive cancers. Neuroscientist 2008;**14**(6):571-83 doi: 10.1177/1073858408320293published Online First: Epub Date]|.
13. House CD, Vaske CJ, Schwartz A, et al. Voltage-gated Na+ channel SCN5A is a key regulator of a gene transcriptional network that controls colon cancer invasion. Cancer Res 2010;**70**(17):6957-67 doi: 10.1158/0008-5472.CAN-10-1169published Online First: Epub Date]|.
14. Grimes JA, Fraser SP, Stephens GJ, et al. Differential expression of voltage-activated Na^+ currents in two prostatic tumour cell lines: contribution to invasiveness in vitro. FEBS Lett 1995;**369**(2-3):290-4
15. Fraser SP, Diss JK, Chioni AM, et al. Voltage-gated sodium channel expression and potentiation of human breast cancer metastasis. Clin Cancer Res 2005;**11**(15):5381-89 doi: 10.1158/1078-0432.CCR-05-0327published Online First: Epub Date]|.
16. Brackenbury WJ, Chioni AM, Diss JK, Djamgoz MB. The neonatal splice variant of Nav1.5 potentiates in vitro metastatic behaviour of MDA-MB-231 human breast cancer cells. Breast Cancer Res. Treat. 2007;**101**(2):149-60
17. Brackenbury WJ, Djamgoz MB. Activity-dependent regulation of voltage-gated Na^+ channel expression in Mat-LyLu rat prostate cancer cell line. J Physiol 2006;**573**(Pt 2):343-56 doi: 10.1113/jphysiol.2006.106906published Online First: Epub Date]|.
18. Brackenbury WJ, Djamgoz MB. Nerve growth factor enhances voltage-gated Na+ channel activity and Transwell migration in Mat-LyLu rat prostate cancer cell line. J Cell Physiol 2007;**210**(3):602-8 doi: 10.1002/jcp.20846published Online First: Epub Date]|.
19. Nelson M, Millican-Slater R, Forrest LC, Brackenbury WJ. The sodium channel beta1 subunit mediates outgrowth of neurite-like processes on breast cancer cells and promotes tumour growth and metastasis. Int J Cancer 2014;**135**(10):2338-51 doi: 10.1002/ijc.28890published Online First: Epub Date]|.
20. Brackenbury WJ, Isom LL. Na Channel beta Subunits: Overachievers of the Ion Channel Family. Frontiers in pharmacology 2011;**2**:53 doi: 10.3389/fphar.2011.00053published Online First: Epub Date]|.
21. Chioni AM, Brackenbury WJ, Calhoun JD, Isom LL, Djamgoz MB. A novel adhesion molecule in human breast cancer cells: voltage-gated Na+ channel beta1 subunit. Int J Biochem Cell Biol 2009;**41**(5):1216-27 doi: 10.1016/j.biocel.2008.11.001published Online First: Epub Date]|.
22. Fraser SP, Salvador V, Manning EA, et al. Contribution of functional voltage-gated Na^+ channel expression to cell behaviors involved in the metastatic cascade in rat prostate cancer: I. lateral motility. J Cell Physiol 2003;**195**(3):479-87
23. Yang M, Kozminski DJ, Wold LA, et al. Therapeutic potential for phenytoin: targeting Na(v)1.5 sodium channels to reduce migration and invasion in metastatic breast cancer. Breast Cancer Res Treat 2012;**134**(2):603-15 doi: 10.1007/s10549-012-2102-9published Online First: Epub Date]|.
24. Brackenbury WJ, Isom LL. Voltage-gated Na+ channels: potential for beta subunits as therapeutic targets. Expert Opin Ther Targets 2008;**12**(9):1191-203 doi: 10.1517/14728222.12.9.1191published Online First: Epub Date]|.

For peer review only

25. Singh G, Driever PH, Sander JW. Cancer risk in people with epilepsy: the role of antiepileptic drugs. Brain 2005;**128**(Pt 1):7-17 doi: 10.1093/brain/awh363published Online First: Epub Date]|.
26. Brisson L, Driffort V, Benoist L, et al. NaV1.5 Na(+) channels allosterically regulate the NHE-1 exchanger and promote the activity of breast cancer cell invadopodia. J Cell Sci 2013;**126**(Pt 21):4835-42 doi: 10.1242/jcs.123901published Online First: Epub Date]|.
27. Ding L, Ellis MJ, Li S, et al. Genome remodelling in a basal-like breast cancer metastasis and xenograft. Nature 2010;**464**(7291):999-1005 doi: 10.1038/nature08989published Online First: Epub Date]|.
28. Grimes JA, Djamgoz MB. Electrophysiological characterization of voltage-gated Na(+) current expressed in the highly metastatic Mat-LyLu cell line of rat prostate cancer. J Cell Physiol 1998;**175**(1):50-8
29. Gillet L, Roger S, Besson P, et al. Voltage-gated Sodium Channel Activity Promotes Cysteine Cathepsin-dependent Invasiveness and Colony Growth of Human Cancer Cells. J Biol Chem 2009;**284**(13):8680-91 doi: M806891200 [pii] 10.1074/jbc.M806891200published Online First: Epub Date]|.
30. Ding Y, Brackenbury WJ, Onganer PU, et al. Epidermal growth factor upregulates motility of Mat-LyLu rat prostate cancer cells partially via voltage-gated Na^+ channel activity. J Cell Physiol 2008;**215**(1):77-81 doi: 10.1002/jcp.21289published Online First: Epub Date]|.
31. Vinogradova Y, Coupland C, Hippisley-Cox J. Exposure to bisphosphonates and risk of gastrointestinal cancers: series of nested case-control studies with QResearch and CPRD data. BMJ (Clinical research ed.) 2013;**346**:f114 doi: 10.1136/bmj.f114published Online First: Epub Date]|.
32. Reeves D, Springate DA, Ashcroft DM, et al. Can analyses of electronic patient records be independently and externally validated? The effect of statins on the mortality of patients with ischaemic heart disease: a cohort study with nested case-control analysis. BMJ open 2014;**4**(4):e004952 doi: 10.1136/bmjopen-2014-004952published Online First: Epub Date]|.
33. *British National Formulary London*: British Medical Association and Royal Pharmaceutical Society of Great Britain, 2013.
34. Gupta GP, Massague J. Cancer metastasis: building a framework. Cell 2006;**127**(4):679-95 doi: 10.1016/j.cell.2006.11.001published Online First: Epub Date]|.
35. IARC. IARC Monographs on the evaluation of carcinogenic risks to humans. Volume 44 Alcohol drinking, 1988.
36. IARC. IARC Monographs on the evaluation of carcinogenic risks to humans. Volume 83 Tobacco smoke and involuntary smoking, 2004.
37. Reeves GK, Pirie K, Beral V, Green J, Spencer E, Bull D. Cancer incidence and mortality in relation to body mass index in the Million Women Study: cohort study. BMJ (Clinical research ed.) 2007;**335**(7630):1134 doi: 10.1136/bmj.39367.495995.AEpublished Online First: Epub Date]|.
38. National Cancer Intelligence Network and Cancer Research UK. Cancer Incidence and Survival by Major Ethnic Group, England 2002-2006., 2009.
39. Coleman RE, Rubens RD. The clinical course of bone metastases from breast cancer. Br J Cancer 1987;**55**(1):61-6
40. Yong M, Jensen AO, Jacobsen JB, Norgaard M, Fryzek JP, Sorensen HT. Survival in breast cancer patients with bone metastases and skeletal-related events: a population-based cohort study in Denmark (1999-2007). Breast Cancer Res Treat 2011;**129**(2):495-503 doi: 10.1007/s10549-011-1475-5published Online First: Epub Date]|.
41. Jensen AO, Jacobsen JB, Norgaard M, Yong M, Fryzek JP, Sorensen HT. Incidence of bone metastases and skeletal-related events in breast cancer patients: a population-based cohort study in Denmark. BMC Cancer

For peer review only

For peer review only - http://bmjopen.bmj.com/site/about/guidelines.xhtml

2011;**11**:29 doi: 10.1186/1471-2407-11-29published Online First: Epub Date]|.
42. Joint Epilepsy Council of the UK and Ireland. Epilepsy prevalence, incidence and other statistics. http://www.epilepsyscotland.org.uk/pdf/Joint_Epilepsy_Council_Prevalence_and_Incidence_September_11_(3).pdf 2011
43. Nicholas JM, Ridsdale L, Richardson MP, Ashworth M, Gulliford MC. Trends in antiepileptic drug utilisation in UK primary care 1993-2008: cohort study using the General Practice Research Database. Seizure : the journal of the British Epilepsy Association 2012;**21**(6):466-70 doi: 10.1016/j.seizure.2012.04.014published Online First: Epub Date]|.
44. Fang MC, Stafford RS, Ruskin JN, Singer DE. National trends in antiarrhythmic and antithrombotic medication use in atrial fibrillation. Arch Intern Med 2004;**164**(1):55-60 doi: 10.1001/archinte.164.1.55published Online First: Epub Date]|.
45. Cancer Research UK. Lifetime risk of cancer. 2014. http://www.cancerresearchuk.org/cancer-info/cancerstats/incidence/risk/statistics-on-the-risk-of-developing-cancer - Lifetime5).
46. von Elm E, Altman DG, Egger M, Pocock SJ, Gotzsche PC, Vandenbroucke JP. Strengthening the Reporting of Observational Studies in Epidemiology (STROBE) statement: guidelines for reporting observational studies. BMJ (Clinical research ed.) 2007;**335**(7624):806-8 doi: 10.1136/bmj.39335.541782.ADpublished Online First: Epub Date]|.

For peer review only

STROBE Statement—checklist of items that should be included in reports of observational studies

	Item No	Recommendation	
**Title and abstract**	1	(*a*) Indicate the study's design with a commonly used term in the title or the abstract	✓
		(*b*) Provide in the abstract an informative and balanced summary of what was done and what was found	✓
**Introduction**			
Background/rationale	2	Explain the scientific background and rationale for the investigation being reported	✓
Objectives	3	State specific objectives, including any prespecified hypotheses	✓
**Methods**			
Study design	4	Present key elements of study design early in the paper	✓
Setting	5	Describe the setting, locations, and relevant dates, including periods of recruitment, exposure, follow-up, and data collection	✓
Participants	6	(*a*) *Cohort study*—Give the eligibility criteria, and the sources and methods of selection of participants. Describe methods of follow-up<br>*Case-control study*—Give the eligibility criteria, and the sources and methods of case ascertainment and control selection. Give the rationale for the choice of cases and controls<br>*Cross-sectional study*—Give the eligibility criteria, and the sources and methods of selection of participants	✓
		(*b*) *Cohort study*—For matched studies, give matching criteria and number of exposed and unexposed<br>*Case-control study*—For matched studies, give matching criteria and the number of controls per case	N/A
Variables	7	Clearly define all outcomes, exposures, predictors, potential confounders, and effect modifiers. Give diagnostic criteria, if applicable	✓
Data sources/ measurement	8*	For each variable of interest, give sources of data and details of methods of assessment (measurement). Describe comparability of assessment methods if there is more than one group	✓
Bias	9	Describe any efforts to address potential sources of bias	✓
Study size	10	Explain how the study size was arrived at	✓
Quantitative variables	11	Explain how quantitative variables were handled in the analyses. If applicable, describe which groupings were chosen and why	✓
Statistical methods	12	(*a*) Describe all statistical methods, including those used to control for confounding	✓
		(*b*) Describe any methods used to examine subgroups and interactions	✓
		(*c*) Explain how missing data were addressed	✓
		(*d*) *Cohort study*—If applicable, explain how loss to follow-up was addressed<br>*Case-control study*—If applicable, explain how matching of cases and controls was addressed<br>*Cross-sectional study*—If applicable, describe analytical methods taking account of sampling strategy	✓
		(*e*) Describe any sensitivity analyses	✓

Continued on next page

**Results**		
Participants	13*	(a) Report numbers of individuals at each stage of study—eg numbers potentially eligible, examined for eligibility, confirmed eligible, included in the study, completing follow-up, and analysed
		(b) Give reasons for non-participation at each stage
		(c) Consider use of a flow diagram
Descriptive data	14*	(a) Give characteristics of study participants (eg demographic, clinical, social) and information on exposures and potential confounders
		(b) Indicate number of participants with missing data for each variable of interest
		(c) *Cohort study*—Summarise follow-up time (eg, average and total amount)
Outcome data	15*	*Cohort study*—Report numbers of outcome events or summary measures over time
		Case-control study—Report numbers in each exposure category, or summary measures of exposure
		Cross-sectional study—Report numbers of outcome events or summary measures
Main results	16	(*a*) Give unadjusted estimates and, if applicable, confounder-adjusted estimates and their precision (eg, 95% confidence interval). Make clear which confounders were adjusted for and why they were included
		(*b*) Report category boundaries when continuous variables were categorized
		(*c*) If relevant, consider translating estimates of relative risk into absolute risk for a meaningful time period
Other analyses	17	Report other analyses done—eg analyses of subgroups and interactions, and sensitivity analyses
**Discussion**		
Key results	18	Summarise key results with reference to study objectives
Limitations	19	Discuss limitations of the study, taking into account sources of potential bias or imprecision. Discuss both direction and magnitude of any potential bias
Interpretation	20	Give a cautious overall interpretation of results considering objectives, limitations, multiplicity of analyses, results from similar studies, and other relevant evidence
Generalisability	21	Discuss the generalisability (external validity) of the study results
**Other information**		
Funding	22	Give the source of funding and the role of the funders for the present study and, if applicable, for the original study on which the present article is based

*Give information separately for cases and controls in case-control studies and, if applicable, for exposed and unexposed groups in cohort and cross-sectional studies.

Note: An Explanation and Elaboration article discusses each checklist item and gives methodological background and published examples of transparent reporting. The STROBE checklist is best used in conjunction with this article (freely available on the Web sites of PLoS Medicine at http://www.plosmedicine.org/, Annals of Internal Medicine at http://www.annals.org/, and Epidemiology at http://www.epidem.com/). Information on the STROBE Initiative is available at www.strobe-statement.org.
