# Supplementary material for: Exposure to sodium channel-inhibiting drugs and cancer survival: protocol for a cohort study using the QResearch primary care database
Source: BMJ Open. 2014 Nov 14;4(11):e006604. doi: 10.1136/bmjopen-2014-006604 (PMC4244419; doi:10.1136/bmjopen-2014-006604)
Supplement: Reviewer comments [file bmjopen-2014-006604.reviewer_comments.pdf]

PEER REVIEW HISTORY

BMJ Open publishes all reviews undertaken for accepted manuscripts. Reviewers are asked to complete a checklist review form (**http://bmjopen.bmj.com/site/about/resources/checklist.pdf**) and are provided with free text boxes to elaborate on their assessment. These free text comments are reproduced below.

ARTICLE DETAILS

**TITLE (PROVISIONAL)**	Exposure to sodium channel-inhibiting drugs and cancer survival: protocol for a cohort study using the QResearch primary care database
**AUTHORS**	Fairhurst, Caroline; Watt, Ian; Martin, Fabiola; Bland, Martin; Brackenbury, William

This article was not externally reviewed at BMJ Open. Protocols that have been independently assessed prior to submission to BMJ Open are usually fast-tracked to publication on the grounds that further substantial changes will not be possible. This independent assessment will usually be external, independent review for both a substantial grant award from a non-commercial or government funder and ethics approval.
